# Forage yield stability of tall fescue genotypes across eight location-year combinations in the United States and Canada

**DOI:** 10.3389/fpls.2026.1870120

**Published:** 2026-07-23

**Authors:** Shiva Om Makaju, Julie Lynn Hansen, Kathleen Glover, Yousef A. Papadopoulos, Virginia Mae Moore, Ali Mekki Missaoui

**Affiliations:** 1 Center for Applied Genetic Technologies, University of Georgia, Athens, GA, United States; 2 Institute of Plant Breeding, Genetics and Genomics, University of Georgia, Athens, GA, United States; 3 Department of Crop and Soil Sciences, University of Georgia, Athens, GA, United States; 4 Plant Breeding and Genetics Section, School of Integrative Plant Science, Ithaca, NY, United States; 5 Kentville Research and Development Centre, Agriculture and Agri-Food Canada, Kentville, NS, Canada; 6 Agriculture and Agri-Food Canada & Dalhousie University, Truro, NS, Canada; 7 School of Integrative Plant Science, Cornell University, Ithaca, NY, United States

**Keywords:** AMMI, BLUP, genotype-by-environment interaction (G×E), genotype plus G×E (GGE), tall fescue, yield weighted stability index

## Abstract

Tall fescue (*Festuca arundinacea* Schreb.) is a cool-season, allohexaploid perennial grass used for forage, soil conservation, and turf. In the United States, it is mainly adapted to the transition zone between the temperate North and mild South, with broad distribution in central and eastern regions. Forage yield depends on genotype, fertilization, and environmental conditions. As the performance of genotypes for tall fescue biomass yield is significantly affected by genotype-by-environment interaction (G×E), we evaluated biomass yield and its stability for 14 tall fescue genotypes across eight location-year combinations in the United States and Canada using 434 observations from a multi-environment trial. We analyzed data using additive main effects and multiplicative interaction (AMMI), genotype plus G×E (GGE) biplot, and restricted maximum likelihood (REML)-based mixed models to estimate best linear unbiased predictions (BLUPs). AMMI analysis revealed significant genotype, environment, and G×E effects (P < 0.001), with the first two interaction principal component axes together explaining a total of 81.8% of the interaction variance. The GGE biplot corroborated environment-specific genotype performance, supported mega-environment delineation, and identified winning genotypes (g2t and k21) within environmental clusters. Mean vs. stability and ideal genotype analyses indicated that k21 and jmx were among the most favorable genotypes for combined yield performance and stability across environments. Mixed-model BLUP estimates and probability-based ranking further supported these findings under partially unbalanced data. For simultaneous selection for productivity and stability, a Yield Weighted Stability Index (YWSI) was calculated by integrating BLUP-predicted yield with AMMI-based stability, providing a composite criterion for comparative evaluation of genotype performance. The YWSI results were consistent with those obtained from the other analytical approaches. The combined use of AMMI, GGE, BLUP, and YWSI provided a comprehensive framework for characterizing G×E patterns and facilitating genotype evaluation across diverse environments.

## 1 Introduction

Tall fescue (*Festuca arundinacea* Schreb.) is a cool-season, allohexaploid (2n = 6x = 42 chromosomes), perennial grass species, with uses such as forage, soil conservation, and turf (Sleper and West, 1996). The major area for tall fescue adaptation in the United States includes a transition zone between the temperate North and mild South, where annual rainfall ranges from 102 to 117 cm, with adaptation occurring mainly from the central to the eastern United States (Burns and Chamblee, 1979). Tall fescue forage yield under these conditions typically ranges from 6 to 15 Mg ha^-1^ yr^-1^, depending on genotype, fertilization, and environmental conditions (Bouton et al., 1993; Burns and Chamblee, 2000). Adaptation areas of tall fescue in the world include Europe, Asia, the United Kingdom, the southern part of Canada, Japan, Australia, New Zealand, the temperate part of Mexico, South America, and Africa (Burns and Chamblee, 1979).

When we grow tall fescue in an environment, we expect to see yield variation among different genotypes. However, in a multi-environment case, the ranking of genotypes may not be consistent across the environments. Additive main effects and multiplicative interaction (AMMI) is a suitable analysis tool for genotype-by-environment interaction (G×E) studies, and it has no requirement for any specific experimental design except for the two-way data structure (Zobel et al., 1988). AMMI integrates analysis of variance (ANOVA) with principal component analysis to partition G×E effects into interaction principal component axes (IPCA1 and IPCA2). The AMMI1 biplot, with mean biomass yield on the X-axis and IPCA1 on the Y-axis, is used to visualize and select genotypes with high yield and high stability (low IPCA1 scores). The AMMI2 biplot, with IPCA1 on the X-axis and IPCA2 on the Y-axis, is used to visualize and identify the most stable genotypes, which are nearest to the biplot origin, and genotypes with strong G×E interaction, which are farthest from the biplot origin. Opposing quadrant placement between a genotype and an environment suggests a negative interaction.

The genotype plus G×E (GGE) biplot focuses on the combined effects of genotype and G×E, using many biplots to assess genotype performance, stability, and possible delineation of mega-environments (Yan et al., 2000; Yan and Kang, 2002). The GGE biplot facilitates identification of high-yielding and stable genotypes and the evaluation of test environments in multi-environment trials. While both AMMI and GGE serve similar objectives in selection, they differ in how they construct and interpret biplots. Mixed-model approaches based on best linear unbiased prediction (BLUP) allow for accurate estimation of genotypic performance while accounting for random effects and form the basis for several derived stability statistics that enhance selection efficiency under unbalanced trial conditions (Olivoto and Lúcio, 2020; Piepho et al., 2008; Resende et al., 2013).

AMMI, GGE, and BLUP-based approaches have been used in the stability analysis of biomass yield of tall fescue and other crops. Multi-environment evaluation is widely used in perennial grass breeding to identify high-yielding and widely adapted genotypes (Kang, 1993; Yan and Kang, 2002). Using AMMI and GGE tools, Saeidnia et al. (2022) were able to identify stable and superior tall fescue genotypes and to delineate two mega-environments and corresponding winner genotypes. Tiwari et al. (2025) applied these tools in evaluating tomato hybrids across nine environments in India, identifying stable, high-yielding hybrids. Multi-environment analysis of tall fescue experimental populations using GGE biplot revealed significant genotype, environment, and G×E effects, with the first two principal components explaining a large proportion of the GGE variation (46.78% and 28.45%) and identifying winner populations in distinct mega-environments (Missaoui et al., 2024). The study further indicated that selection under grazing pressure in stress environments improved yield stability, although intrinsic yield potential remained genotype dependent (Missaoui et al., 2024; Yan and Kang, 2002). GGE biplot was successfully utilized by Lima et al. (2023) to select recommended corn hybrids for different environments of the North and Midwest regions of Brazil.

It is important to integrate biomass yield performance with stability analyses when evaluating tall fescue germplasm across diverse environments, as G×E often complicates cultivar selection because genotypes that perform well in one environment may not maintain similar performance across other environments (Kang, 1993). Selection of genotypes solely based on yield may overlook potential crossover G×E, whereas simultaneous selection for yield and stability may face reluctance in adoption due to concerns about potential yield reduction (Kang, 1993). Therefore, selection criteria should prioritize yield performance while also incorporating yield stability across a wide range of environments. Integrating both yield and stability into the selection process has therefore been widely recommended to improve cultivar evaluation and reduce the risk of recommending genotypes that perform inconsistently under farmer conditions (Kang, 1993).

In perennial switchgrass, BLUP-based drought adaptation index, AMMI model, and GGE biplots were utilized in the identification and selection of genotypes that exhibit both high yield and stable biomass yield performance across diverse environments (Makaju et al., 2026). However, many previous studies have relied on a single analysis tool, limited environmental coverage, or short evaluation periods. Forage breeding presents additional challenges because factors such as perennial growth habit, mating system, and evaluation context (e.g., spaced plants vs. sward plots) can influence trait expression and reduce the efficiency of selection based solely on phenotypic performance (Resende et al., 2013). Differences in plant competition and density between these evaluation systems may also result in variable correlations between spaced-plant and sward-plot performance for complex traits, such as forage yield (Casler and Brummer, 2008).

BLUP-based approaches can improve selection efficiency, particularly for traits with low heritability (<0.30), and are well-suited for analyzing unbalanced multi-environment datasets commonly encountered in forage evaluation programs (Resende et al., 2013). Multi-environment trials (MET) are widely used in plant breeding programs to evaluate genotype performance across diverse environments and to identify high-yielding and stable genotypes. However, the presence of G×E complicates the identification of superior and stable genotypes, requiring robust statistical approaches to interpret genotype performance across environments. To address this challenge, several statistical approaches, such as AMMI and GGE biplot analyses, have been widely applied to evaluate genotype stability and adaptability in MET data (Van Eeuwijk et al., 2016; Yan and Kang, 2002). Recently, integrating environmental characterization into MET analyses has improved the understanding of G×E and genotype adaptation. Envirotyping-based approaches characterize environmental conditions using climatic and agronomic variables to better interpret genotype performance across environments (Resende et al., 2021). These approaches are increasingly used to improve the interpretation of G×E and genotype adaptation. Such analyses have also been used to dissect yield stability and distinguish stable versus environment-specific genomic regions underlying genotype performance across contrasting environments (Corlouer et al., 2024).

Statistical modeling of G×E has been extensively reviewed in plant breeding literature, highlighting the importance of integrating multiple analytical approaches for reliable genotype evaluation (Van Eeuwijk et al., 2016). Therefore, the objective of this study was to evaluate G×E and yield stability of tall fescue genotypes across multiple years and locations, and to identify superior genotypes combining high biomass yield with stable performance across diverse environments.

## 2 Materials and methods

### 2.1 Experimental sites and plant materials

**Figure 1:**
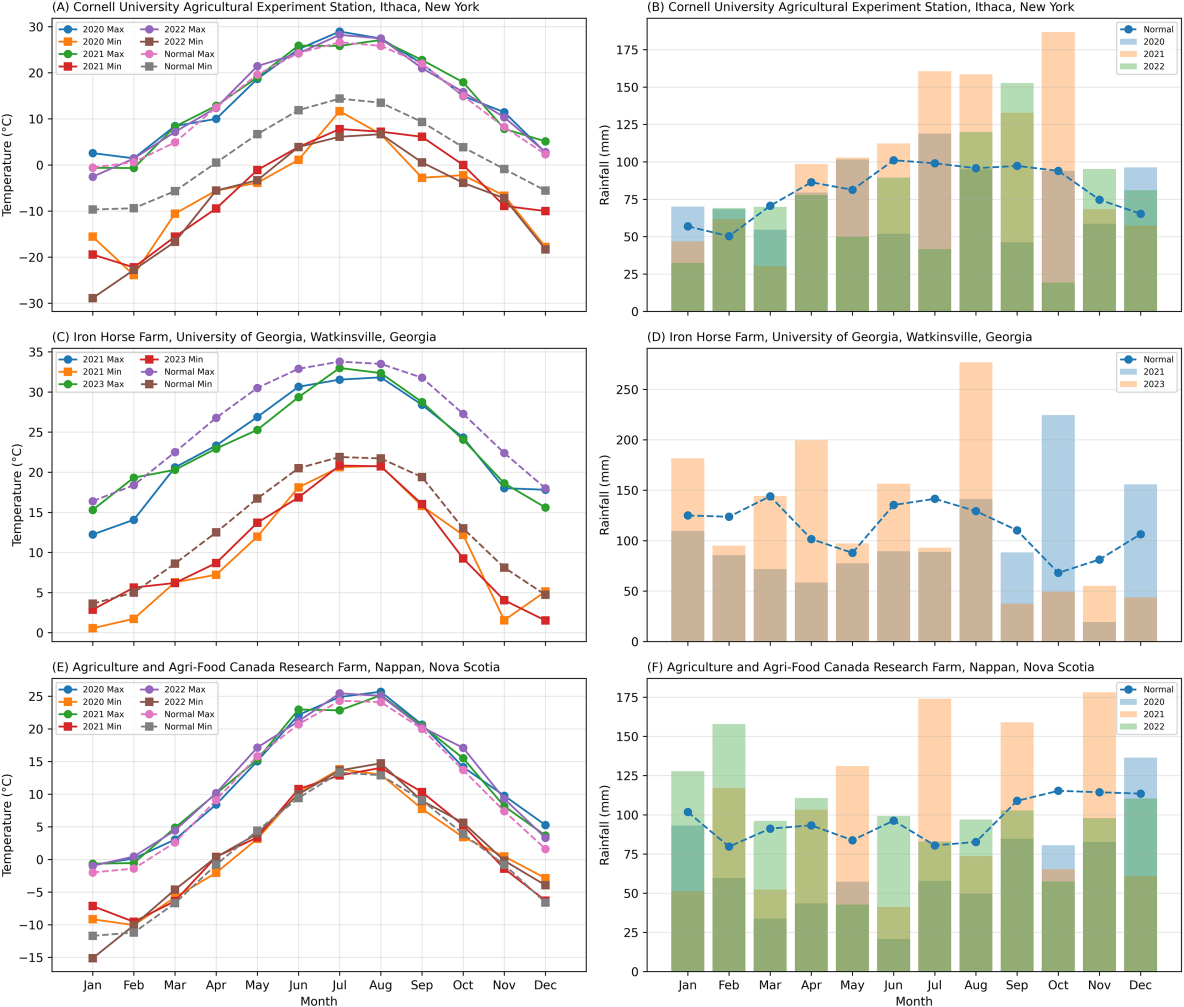
Monthly average daily maximum and minimum temperatures (°C) and monthly total precipitation (mm) during the experimental period and long-term climate normals at three study sites. For each site, two panels are presented: the first shows monthly average maximum and minimum temperatures (°C) and the second shows monthly total precipitation (mm). Panels **(A)** (temperature) and **(B)** (precipitation) are for Cornell University Agricultural Experiment Station, Ithaca, New York. Panels **(C)** (temperature) and **(D)** (precipitation) are for Iron Horse Farm, University of Georgia, Watkinsville, Georgia. Panels **(E)** (temperature) and **(F)** (precipitation) are for the Agriculture and Agri-Food Canada Research Farm, Nappan, Nova Scotia. Long-term climate normals are shown for comparison with observed weather conditions during the study years. Note: Weather data for Iron Horse Farm in Watkinsville, Georgia, were sourced from the University of Georgia Weather Network. Data for the Cornell University Agricultural Experiment Station in Ithaca, New York, were obtained from Cornell University weather records. Data for the Agriculture and Agri-Food Canada Research Farm in Nappan, Nova Scotia, were collected from Environment and Climate Change Canada historical climate records. Long-term climate normals for Cornell and Nappan correspond to the 1991–2020 climatological normal period. For Iron Horse Farm, long-term averages were calculated using the complete historical weather records available from the University of Georgia Weather Network.

The experimental sites were Iron Horse Farm, University of Georgia, Watkinsville, Georgia, USA (33.716531, −83.312516); Cornell University Agricultural Experiment Station, Ithaca, New York, USA (42.443722, −76.442167); and Agriculture and Agri-Food Canada Research Farm, Nappan, Nova Scotia, Canada (45.758771, −64.238644). Multi-environment field trials were conducted at these locations from 2020 to 2023 to evaluate G×E and yield stability in tall fescue. **Figure 1** shows monthly average daily maximum and minimum temperatures (°C) and monthly total precipitation (mm) during the experimental period and long-term climate normals at the three sites. Monthly means of daily maximum and minimum temperatures and monthly total precipitation were calculated and visualized using Python version 3.12.3 with the pandas and Matplotlib packages. The soil at the University of Georgia Iron Horse Farm experimental site was classified as Wickham sandy loam (WkB), characterized by 2–6% slopes and rare flooding, according to the USDA Web Soil Survey. The soil at the Cornell University experimental site in Ithaca, New York, was classified as Rhinebeck silt loam with 2–6% slopes. The soil at the Agriculture and Agri-Food Canada Research Farm in Nappan, Nova Scotia, was classified as Pugwash 52, a well-drained coarse loam mineral soil.

A total of 14 genotypes were evaluated in eight environments, each representing a specific combination of location and year. Environments were identified using a three-character code, where the first letter denotes the location (C = Cornell University Agricultural Experiment Station, Ithaca, New York; I = Iron Horse Farm, University of Georgia, Watkinsville, Georgia; N = Agriculture and Agri-Food Canada Research Farm, Nappan, Nova Scotia) and the last two digits indicate the harvest year. Accordingly, the eight environments were C20, C21, C22, I21, I23, N20, N21, and N22. The genotypes evaluated, along with their abbreviations, were GA 5 (g05), GA 29 (g29), GA 186 (g86), GALA 1502T (g2t), GALA 1503T (g3t), Jesup MaxQ (jmx), Jesup Minus (jmn), Texoma MaxQ II (tma), KY 31+ (k31), KYFA 1405 (k05), KYFA 9611 (k11), KYFA 9821/AR584TF (k21), Lacefield MaxQ II (lcm), and Kokanee (kkn). The first five genotypes were experimental breeding populations, whereas the remaining nine were commercial cultivars or standard checks.

### 2.2 Field establishment and management

The experimental design for each environment used a randomized complete block design (RCBD), with genotypes randomized within each replication (block). Each environment evaluated the same 14 tall fescue genotypes in similarly sized, replicated plots. However, the number of replications per environment varied: Cornell environments (C20, C21, and C22) had four replications per genotype, Iron Horse Farm environments (I21 and I23) had five; Nappan environments (N20, N21, and N22) had three. Thus, while plot size and management were consistent, the trial was partially unbalanced due to the differing numbers of replications among environments (**Table 1**). Both fixed-effects ANOVA and mixed-model analysis accounted for this experimental structure when estimating genotypic effects and stability metrics.

**Table 1:** Environment-by-replication trial structure and number of genotypes across locations and years.

Location	Year	Environment	Rep 1	Rep 2	Rep 3	Rep 4	Rep 5
Cornell	2020	C20	14	14	14	14	0
Cornell	2021	C21	14	14	14	14	0
Cornell	2022	C22	14	14	14	14	0
Iron Horse Farm	2021	I21	14	14	14	14	14
Iron Horse Farm	2023	I23	14	14	14	14	14
Nova Scotia	2020	N20	14	14	14	0	0
Nova Scotia	2021	N21	14	14	14	0	0
Nova Scotia	2022	N22	14	14	14	0	0

Values represent the number of genotypes evaluated in each replication within each environment.

At all locations, tall fescue was established in plots measuring 1.52 m wide by 4.57 m long. Seeding was performed at rates consistent with agronomic recommendations (ca. 20.2 kg ha^-1^) using seed in an envelope consisting of ca. 16 g plot^-1^. The seed rate for Jesup Minus was doubled (ca. 30 g plot^-1^) to compensate for reduced germination based on prior seed quality assessments. Seed was sown following standard field preparation practices, including cultipacking before planting to promote uniform seed-soil contact. Weed management was implemented using pre- or post-emergence herbicide applications as appropriate for each site, and standard agronomic practices were followed throughout the growing season to maintain stand health and minimize non-treatment variation. Following seeding, Quinstar herbicide was applied at 1.12 kg ha^-1^ in combination with methylated seed oil at 2.34 L ha^-1^.

### 2.3 Biomass harvest

Biomass harvest timing and frequency varied among locations, reflecting differences in climate, growing season length, and locally recommended management practices typical for perennial forage systems. Consequently, the number of harvests differed among environments. For each plot, fresh biomass samples were measured at each harvest, and biomass samples were dried to constant weight at 55 °C for five days to determine dry matter content. Plot-level fresh biomass was converted to dry biomass yield on a per-hectare basis (Mg ha^-1^) for each harvest. Annual biomass yield was then calculated as the sum of dry biomass yield across all harvests within each location-year combination. Because each location-year combination was treated as a distinct environment in the analysis, variation arising from differences in harvest frequency was incorporated into the environmental effect.

### 2.4 Statistical analysis

All statistical analyses were conducted using R version 4.4.1 within a Jupyter Notebook environment. MET analyses were performed using the metan package (version 1.19.0) (Olivoto and Lúcio, 2020). The dataset consisted of 14 tall fescue genotypes evaluated across eight environments, with three to five replications per environment. Specifically, five, four, and three replications were associated with environments at Iron Horse Farm, Cornell University, and Nova Scotia, respectively (**Table 1**), resulting in a total of 434 observations.

Before statistical analysis, the dataset was inspected for missing values, distributional properties, and potential outliers. As no influential outliers were identified, all 434 observations were retained for analysis. Exploratory visualizations of yield distributions by environment were used to assess heterogeneity across environments (**Supplementary Figure S1**).

#### 2.4.1 Analysis of variance and genotype mean comparison

A fixed-effects ANOVA was performed to evaluate the effects of genotype, environment, and G×E on biomass yield. For this, the model included genotype and environment as fixed factors, with replications treated as nested within environments. Significance of main and interaction effects was assessed using F-tests at the 5% probability level (α = 0.05). For summary comparison, overall genotype estimated marginal means across environments were computed, and pairwise comparisons among genotypes were performed using Tukey’s honestly significant difference (HSD) test at α = 0.05.

#### 2.4.2 AMMI model

To assess G×E and yield stability, the AMMI model was applied. The AMMI model combines ANOVA for the additive effects of genotypes and environments with PCA of the interaction residuals (Gauch Jr, 1988). The AMMI model was applied following the original formulation by Gauch (1988):


$$Y_{ge}=µ+\alpha_{g}+\beta_{e}+\sum_{n=1}^{N}\lambda_{n}\gamma_{gn}\delta_{en}+\theta_{ge}$$

where *Y_ge_* represents yield of genotype g in environment e; *μ* represents the grand mean; *α_g_* represents the difference of the genotype mean minus the grand mean; *β_e_* represents the difference of the environment mean minus the grand mean; λ*_n_* represents the eigenvalue of PCA axis n; N represents the number of PCA axes retained in the model; γ*_gn_* represents the genotype PCA score for PCA axis n; δ*_en_* represents the environment PCA score for PCA axis n; *θ_ge_* represents the residual. In the AMMI framework, genotype and environment main effects are modeled additively, and the G×E is decomposed into IPCAs through singular value decomposition of the interaction matrix.

#### 2.4.3 GGE biplot

The GGE biplot model, originally formulated by (Yan et al., 2000), was used to visualize the genotype main effect and the G×E effect together. The identification of superior genotypes, delineation of mega-environments, and determination of the best genotype(s) for each mega-environment using GGE biplots are useful for effective germplasm selection. In the GGE biplot framework, biplots were constructed using environment-centered yield data, and PCA was applied to extract the first two principal components (PC1 and PC2), which capture most of the variation attributable to genotype and G×E effects (Frutos et al., 2014; Yan et al., 2000).

#### 2.4.4 Mixed-effects model and biomass yield BLUP

A linear mixed-effects model was fitted using restricted maximum likelihood (REML) to estimate biomass yield BLUPs for genotype performance across environments. In this model, genotype and G×E were treated as random effects, whereas environments and replications nested within environments were treated as fixed effects. Mixed-model approaches are widely recommended for the MET analysis because they provide efficient estimation of genotype performance under unbalanced data structures (Piepho et al., 2012). Variance components were estimated for genotypic and G×E effects, and their significance was assessed using likelihood ratio tests (LRTs) (**Table 2**). Genotype BLUPs for annual biomass yield were evaluated for overall performance and ranked across environments, as well as using ranked probability-based BLUPs. The probability-based ranking complemented BLUP-based mean estimates to assess the likelihood of superior genotype performance across environments. Model adequacy was evaluated through residual diagnostics, including the distribution of residuals and Q-Q plots (**Supplementary Figures S2**, **S3**), which indicated that residuals were approximately normally distributed with no substantial deviation from normality.

**Table 2:** Variance components and likelihood ratio tests (LRT) for annual biomass yield.

Effect	Variance	% of total	LRT	df	P-value
Genotype	0.10	7.00	5.86	1.00	0.02
G×E	0.29	22.00	25.52	1.00	<0.001
Residual	0.95	71.00			

#### 2.4.5 Stability parameters and yield weighted stability index

The Shukla’s stability variance (σ²_i_), Wricke’s ecovalence (Wi), AMMI stability index (ASI), and AMMI stability value (ASV) were calculated to quantify genotype contribution to G×E, where lower values indicate greater stability (**Table 3**). Shukla’s stability variance partitions the G×E variance into genotype-specific components, providing a measure of stability in which smaller variance values indicate more consistent genotype performance across environments (Shukla, 1972). Based on estimated genotype BLUPs from a linear mixed-effects model fitted using REML, the weighted average of absolute scores from BLUP (WAASB), harmonic mean of genotypic values (HMGV), relative performance of genotypic values (RPGV), harmonic mean of the relative performance of genotypic values (HMRPGV), and Lin and Binns’ superiority index (Pi) were calculated; lower values indicate superior performance (**Table 3**). Genotypic performance across environments was predicted using BLUP within a mixed linear model framework with variance components estimated by REML. BLUP improves the accuracy of genotypic value estimation through shrinkage and efficiently handles unbalanced multi-environment trial data, enabling a more reliable selection of superior genotypes (Piepho et al., 2008). WAASB is a stability measure derived from the singular value decomposition of the matrix of BLUP-estimated G×E effects, allowing stability assessment within a mixed-model framework (Olivoto et al., 2019). Implementation of these stability metrics in multi-environment trial analysis is available through the metan package (Olivoto and Lúcio, 2020).

**Table 3:** Yield performance and stability parameters of 14 tall fescue genotypes evaluated across eight environments.

Genotype	Yield (Mg ha^-1^)	Yield rank	CV of yield (%)	Shukla (σ²_i_)	Ecovalence (Wi)	ASI	ASI rank	ASV	WAASB	WAASB rank	HMGV	RPGV	HMRPGV	Pi	Pi rank
g05	9.63	10	41	0.072	3.49	0.11	4	0.37	0.133	4	8.31	1.00	1.00	0.778	7
g29	9.83	5	43	0.094	4.15	0.14	5	0.47	0.153	5	8.39	1.01	1.01	0.648	6
g2t	10.22	1	39	0.585	18.88	0.35	10	1.14	0.386	12	8.87	1.06	1.06	0.591	5
g3t	9.28	12	41	0.213	7.73	0.21	7	0.68	0.216	9	8.08	0.97	0.97	1.542	12
g86	9.83	6	41	0.552	17.90	0.24	9	0.77	0.191	6	8.44	1.01	1.01	0.578	4
jmn	9.64	9	43	1.072	33.51	0.40	12	1.30	0.322	10	8.30	1.00	1.00	1.445	11
jmx	10.05	3	39	0.11	4.64	0.15	6	0.47	0.207	8	8.74	1.04	1.04	0.391	1
k05	9.62	11	42	0.119	4.92	0.06	2	0.20	0.081	2	8.32	1.00	1.00	1.021	10
k11	9.69	8	41	0.078	3.67	0.08	3	0.27	0.113	3	8.34	1.00	1.00	0.877	9
k21	10.20	2	42	0.704	22.45	0.38	11	1.23	0.339	11	8.74	1.05	1.04	0.432	2
k31	8.92	13	41	1.65	50.82	0.69	13	2.25	0.622	13	7.76	0.94	0.93	2.548	13
kkn	8.89	14	50	1.963	60.21	0.76	14	2.47	0.713	14	7.25	0.91	0.89	2.743	14
lcm	9.71	7	44	0.129	5.22	0.04	1	0.12	0.077	1	8.28	1.00	1.00	0.845	8
tma	9.97	4	42	0.148	5.78	0.21	8	0.68	0.200	7	8.54	1.02	1.02	0.478	3

Yield, biomass yield (Mg ha^-1^); CV (%), coefficient of variation; σ²_i_, Shukla’s stability variance; Wi, Wricke’s ecovalence; ASI, AMMI stability index; ASV, AMMI stability value; WAASB, weighted average of absolute scores from BLUP; HMGV, harmonic mean of genotypic values; RPGV, relative performance of genotypic values; HMRPGV, harmonic mean of the relative performance of genotypic values; Superiority (Pi), Lin and Binns’ superiority index.

To facilitate simultaneous selection for yield and stability, a Yield Weighted Stability Index (YWSI) was calculated by integrating environment-specific BLUP-predicted yield with AMMI-derived stability, expressed as ASV, following the approach proposed by Irfan et al. (2026). Specifically, genotypes were ranked for BLUP-predicted biomass yield within each environment (C20, C21, C22, I21, I23, N20, N21, and N22) and for ASV. The YWSI was calculated as the sum of these nine ranks. The AMMI model partitions variation into genotype main effects, environment main effects, and G×E interaction effects, allowing stability to be quantified from interaction principal components (Gauch Jr, 2006). Thus, the YWSI provides a composite measure of overall genotype performance by jointly considering productivity and stability, where lower index values indicate superior combined yield and stability.

## 3 Results

### 3.1 Trial structure and yield variation across environments

The multi-environment trial consisted of 14 tall fescue genotypes evaluated across eight location-year combinations with three to five replications per environment (**Table 1**). The dataset comprised 434 total observations, reflecting the partially unbalanced replication structure across sites. Substantial variation in biomass yield was observed across environments. Across all 434 observations, annual biomass yield ranged from 2.48 to 19.38 Mg ha^-1^, with a mean of 9.10 Mg ha^-1^ and a median of 7.78 Mg ha^-1^. The dataset showed relatively high variability (coefficient of variation (CV) = 42%), with a standard deviation of 3.85 Mg ha^-1^ and standard error of 0.18 Mg ha^-1^. The 95% confidence interval of the mean was ±0.36 Mg ha^-1^, indicating substantial variation in yield values across environments. Yield distributions within individual environments varied in range and variability, reflecting environmental heterogeneity across the test sites (**Supplementary Figure 1**).

### 3.2 Fixed-effects analysis of variance

The fixed-effects analysis of variance showed significant effects of genotype, environment, and G×E interaction on biomass yield (**Table 4**). This analysis represents the traditional ANOVA framework used here as a preliminary step in MET to partition the main sources of variation before AMMI and mixed-model analyses. The environmental effect accounted for the largest proportion of the total variation, indicating strong differences among test location-year combinations. Genotypic differences were also significant, implying the presence of genetic variability for yield among the evaluated tall fescue entries. Because the G×E interaction was significant, genotype biomass yield performance was expected to vary across environments.

**Table 4:** Fixed-effects analysis of variance (ANOVA) for annual biomass yield of 14 tall fescue genotypes evaluated across eight environments.

Source of variation	df	Sum of squares (SS)	Mean SS	F value	Pr(>F)
Genotype (G)	13	69.39	5.34	5.60	3.83E-09
Environment (E)	7	5698.36	814.05	853.58	1.79E-193
Rep(Environment)	23	179.49	7.80	8.18	8.76E-21
G×E	91	188.55	2.07	2.17	4.99E-07
Residual	299	285.15	0.95		

Rep(Environment), replication nested within environment; G×E, interaction; DF, degrees of freedom.

### 3.3 AMMI analysis of variance and interaction decomposition

The AMMI analysis of variance indicated that genotype, environment, and G×E had a significant effect on total biomass yield variation (P < 0.001; **Table 5**). As expected in perennial forage such as tall fescue, environmental effects accounted for the largest proportion of total variation [Sum of Squares (SS) = 5698.36], reflecting differences in climate, management, and harvest frequency among location-year combinations. The proportion of total variation due to genotypic effects was SS = 69.39, and due to the G×E interaction was SS = 188.55, confirming differential genotype responses across environments. Decomposition of the interaction sum of squares showed that IPCA1 and IPCA2 explained 51.2% and 30.5% of the interaction variance, respectively. Genotype and environmental means, along with their corresponding IPCA scores, are provided in **Supplementary Table S1**.

**Table 5:** AMMI analysis of variance (ANOVA) for yield.

Source of variation	df	Sum of squares (SS)	Mean SS	F-value	P-value	Proportion
Environment (E)	7	5698.36	814.05	104.31	0	
Rep(Environment)	23	179.49	7.80	8.18	0	
Genotype (G)	13	69.39	5.34	5.60	0	
G×E	91	188.55	2.07	2.17	0	
IPCA1	19	124.65	6.56	6.88	0.00	51.20
IPCA2	17	74.33	4.37	4.58	0.00	30.50
Residuals	299	285.15	0.95			
Total	524	6664.32	12.72			

#### 3.3.1 Genotype and environment mean yield against IPCA1

**Figure 2:**
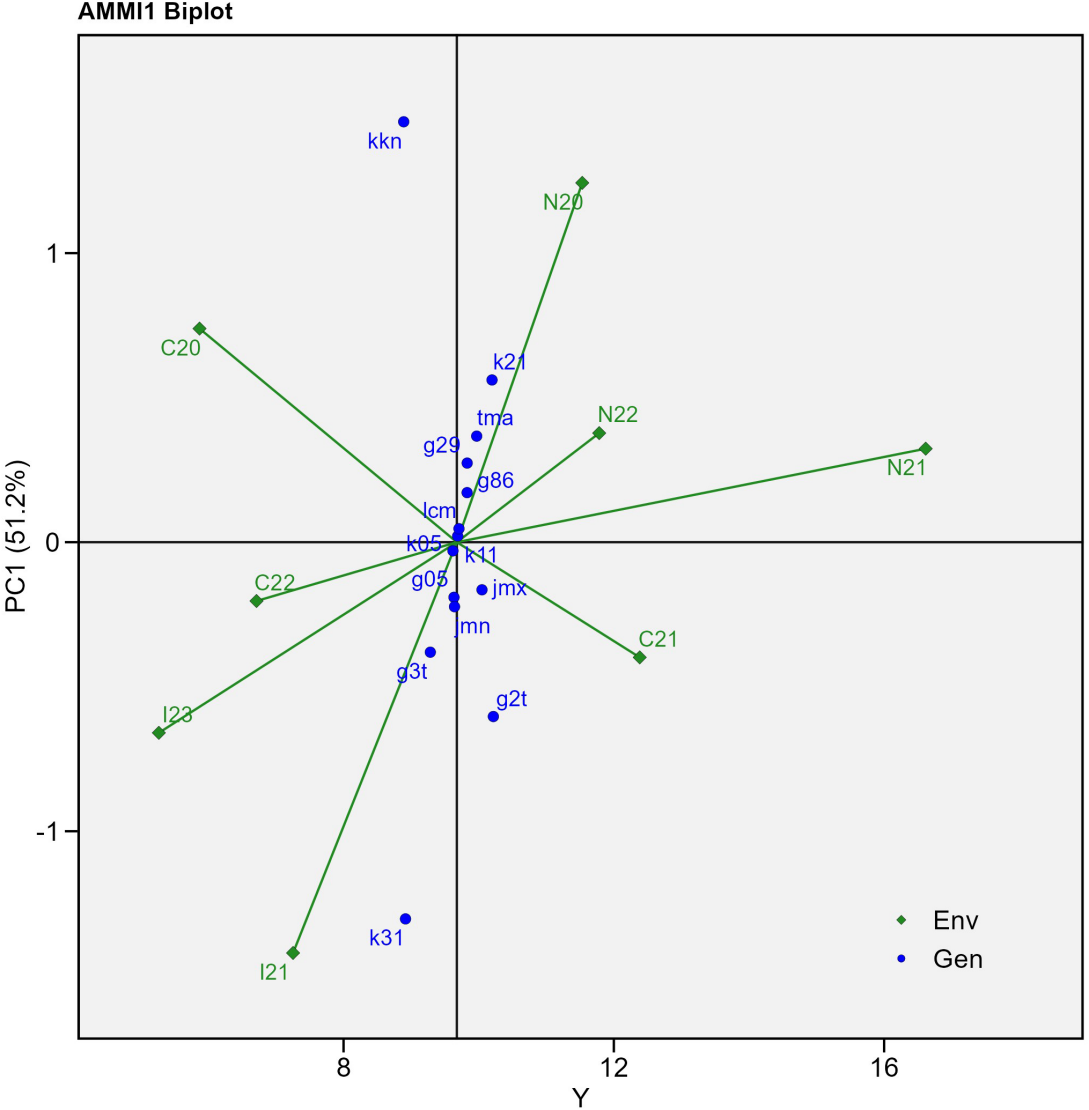
Genotype-by-environment mean yield (Y) against IPCA1 plot. AMMI biplot showing genotype-by-environment mean yield plotted against the first interaction principal component axis (IPCA1). Genotypes and environments closer to the origin exhibit greater stability, whereas those farther from the origin contribute more strongly to G×E.

**Figure 2** presents the AMMI1 biplot with genotype and environment mean yield (Y) on the x-axis and the IPCA1 on the y-axis, which explained 51.2% of the G×E (**Figure 2**; **Table 5**). Genotypes located near the horizontal axis (IPCA1 ≈ 0), such as g05, k05, lcm, and k11 exhibited relatively small interaction effects and greater stability across environments. In contrast, genotypes positioned farther from the axis, including kkn and k31, showed larger interaction effects, indicating stronger specific responses to certain environments. Genotypes g2t, jmx, and k31 that are located towards the right side of the biplot ordinate have higher mean yield; similarly, environments N21 and N20 exhibited higher mean yield, whereas I23 and I21 were associated with lower mean yield. Environments positioned farther from the horizontal axis, particularly N20 and I21, contributed more strongly to G×E. The distribution of genotypes above and below the axis further indicates differential responses across environments, supporting the presence of crossover interaction. Mean yield and AMMI-based stability parameters are presented in **Table 3**.

#### 3.3.2 AMMI biplot for yield across environments and IPCAs

**Figure 3:**
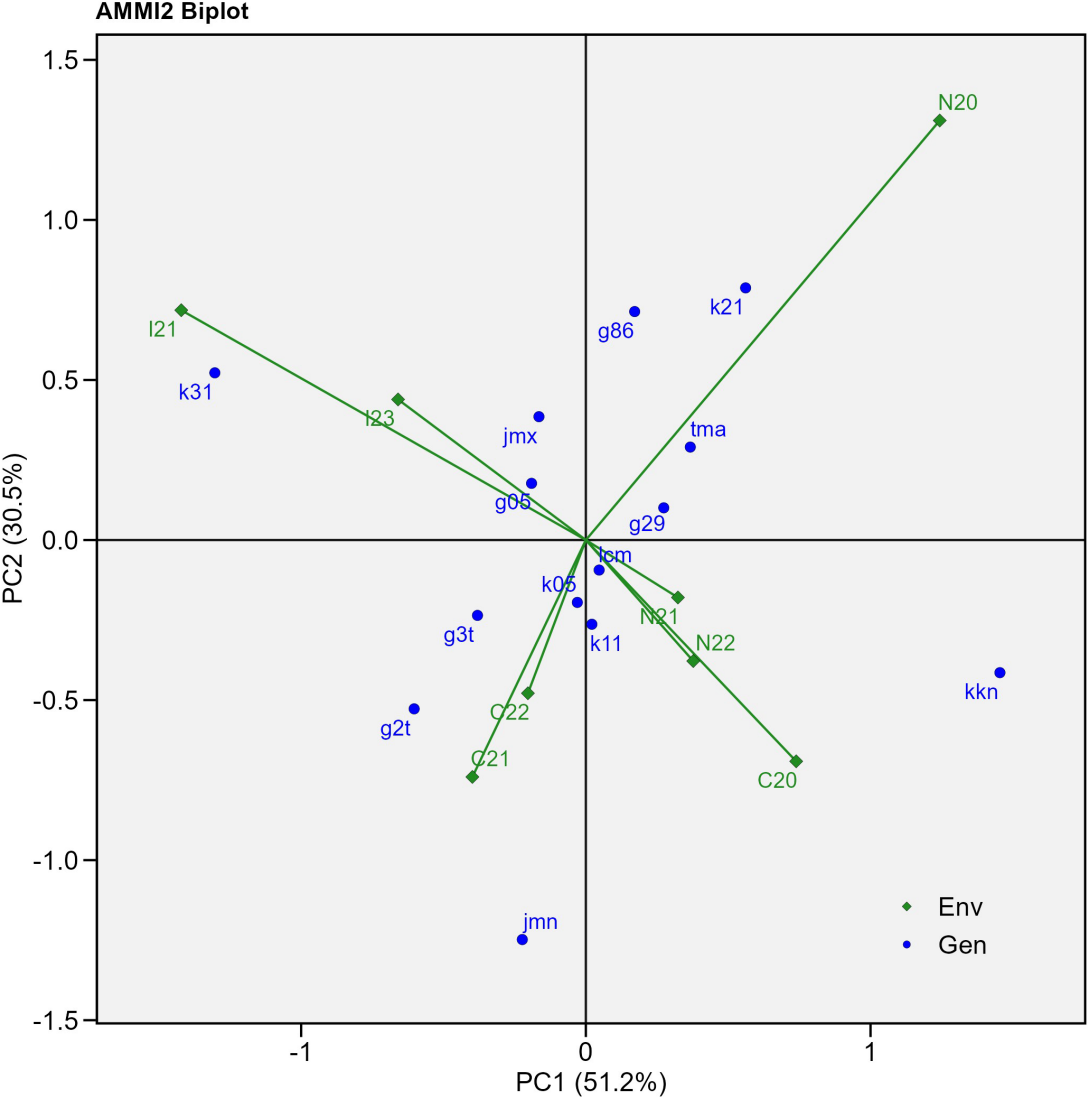
AMMI biplot for yield across environments. AMMI biplot showing genotype and environment scores for the first two interaction principal component axes (IPCA1 and IPCA2), explaining interaction relationships and specific adaptation patterns.

**Figure 3** presents the AMMI2 biplot where IPCA1 and IPCA2 accounted together for 81.8% of the total G×E, indicating that the first two axes adequately captured the majority of the interaction effects, and that is the reason that the interpretation in general focused primarily on IPCA1 and IPCA2. The relatively large environmental sum of squares compared to genotypic and interaction components highlights the substantial environmental heterogeneity across the eight test environments. The significant interaction component indicates the presence of crossover responses among genotypes.

Based on **Figure 3**, genotypes and environments located near the origin exhibited relatively small interaction effects and greater stability across environments. In contrast, those positioned farther from the origin contributed more strongly to G×E interaction. The figure showed that environment N20 was positioned far from the origin along both axes, indicating strong discriminating ability and substantial interaction effects. Similarly, genotype kkn showed large interaction effects, suggesting specific adaptation rather than broad stability. The separation of genotypes across different quadrants indicates crossover interaction, where genotype rankings varied among environments. Environments positioned in similar directions (e.g., N21 and N22) showed comparable interaction patterns, whereas those located in opposite directions indicated contrasting genotype responses.

### 3.4 GGE biplot analysis

**Figure 4:**
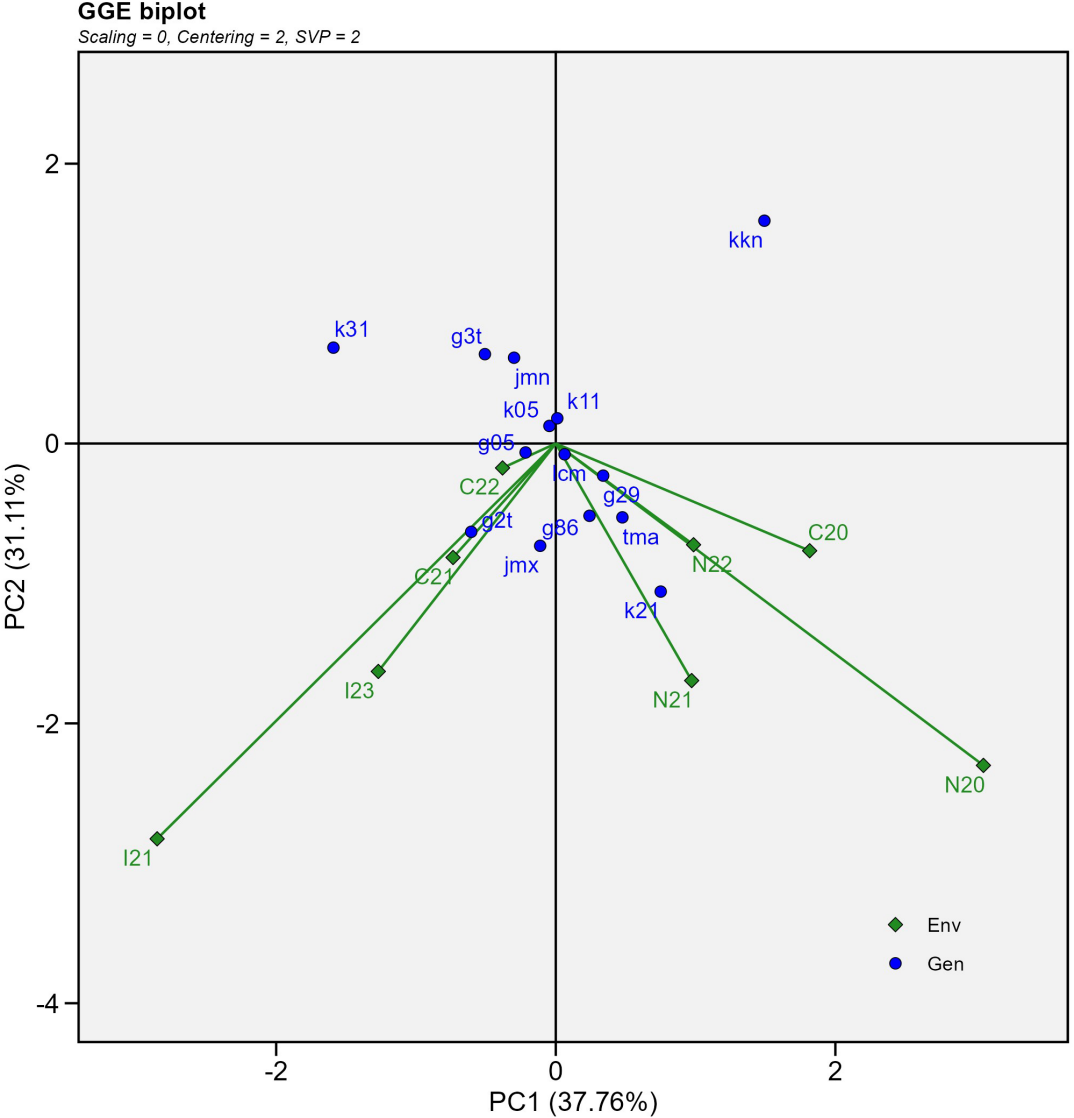
GGE biplot for yield across environments. GGE biplot showing genotype and environment scores for the first two principal component axes (PC1 and PC2). The biplot shows genotype performance and G×E patterns, with genotypes and environments farther from the origin contributing more strongly to interaction effects.

Based on the GGE biplot, PC1 and PC2 explained 37.76% and 31.11% of total genotype plus G×E variation, respectively (**Figure 4**). The two PCs explained the majority of GGE effects across the eight environments. In the GGE biplot, genotypes located near the origin exhibited comparatively stable performance across environments, while the genotypes located farther from the biplot origin contributed more strongly to G×E. In **Figure 4**, genotypes such as g05, k05, and lcm, being located closer to the origin, were comparatively stable performers, while genotype kkn, being positioned at greater distances from the origin, indicated strong discriminating ability and high interaction effects. The placement of genotypes across different quadrants reflects crossover interaction, where genotype rankings varied among environments. The pattern is consistent with the AMMI ANOVA (**Table 5**).

#### 3.4.1 Which-won-where biplot and mega-environment identification

**Figure 5:**
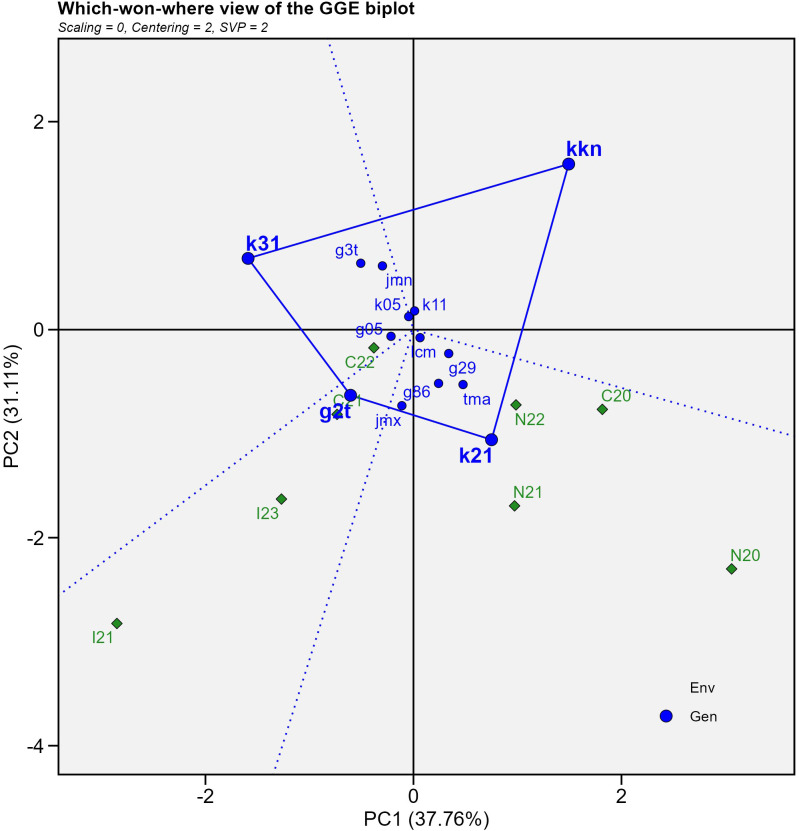
Which-won-where GGE biplot for yield. Which-won-where GGE biplot showing genotype performance across environments based on the first two principal components. The genotypes at the vertex represent those with superior performance in specific environment groups, defined as potential mega-environments.

The which-won-where view of the GGE biplot (**Figure 5**) revealed distinct polygon sectors and corresponding vertex genotypes that are winners in those sectors or mega-environments, while also indicating the presence of crossover G×E. The genotypes located at the vertices of the polygon - g2t, k21, kkn, and k31 were the most responsive and showed superior performance in specific subsets (mega-environments) of environments. In **Figure 5**, the two adjacent dotted lines (perpendiculars from the biplot origin to the polygon sides) form a sector, and the vertex genotype is the best performer in that mega-environment. Environments falling within the same sector shared the same winning genotype. For example, environments N20, N21, N22, and C20 were located within the sector defined by genotype k21, indicating its superior performance in those environments. Similarly, I21, I23, and C21 were positioned in the sector associated with genotype g2t, suggesting it as the winning genotype in those environments. Similarly, k31 is the winner in the C22 environment. The presence of multiple sectors confirms the existence of crossover interaction, where different genotypes performed best in different environment groups (mega-environments). The mega-environment delineation helps in selecting genotypes according to specific environmental clusters rather than relying solely on overall mean performance.

#### 3.4.2 Mean biomass yield and stability of genotypes

**Figure 6:**
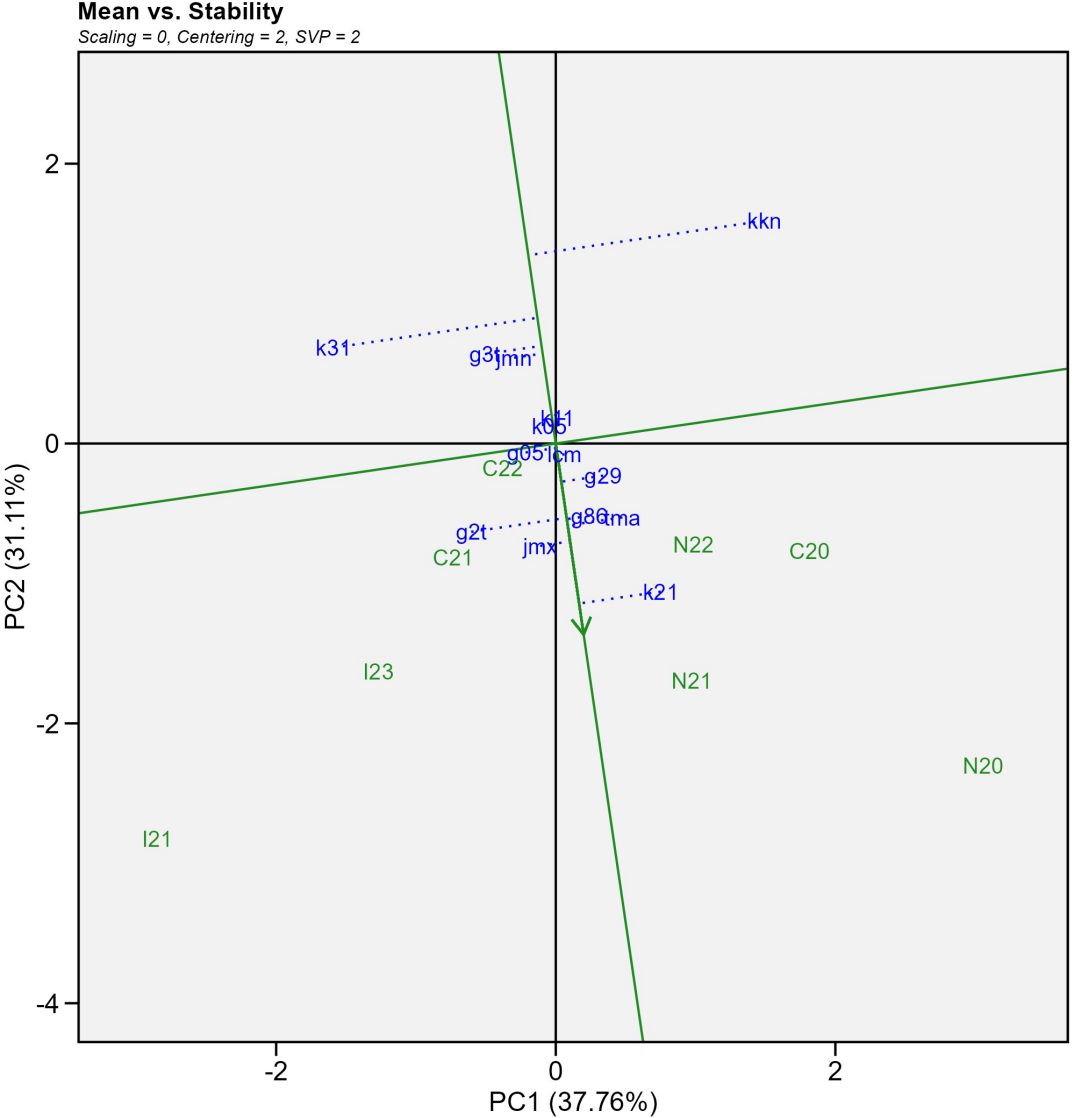
Mean vs. stability GGE biplot for yield. Mean vs. stability GGE biplot showing genotype performance and stability across environments. Genotypes with higher mean yield and shorter projection onto the stability axis are considered better for broad adaptation.

**Figure 6** ranked genotypes based on both average yield and stability across environments. The average environment axis (AEA) represents mean performance, with genotypes positioned further along the positive direction exhibiting higher mean yield (towards the direction of the arrowhead). Stability is indicated by the distance of a genotype from the AEA, where shorter projections reflect greater stability. Genotypes positioned farther from the axis, including k21, jmx, tma, g86, and g2t, had comparatively higher mean biomass yield. Genotypes located near the origin and closer to the AEA, such as g05, k05, and lcm, exhibited comparatively stable performance across environments. Genotypes kkn and k31 had higher perpendicular distance (projections) from the AEA, indicating higher associated variation and the least stability values. Genotypes g86 and lcm had higher stability while having greater than average biomass yield as well.

#### 3.4.3 Discriminating ability and representativeness of test environments

**Figure 7:**
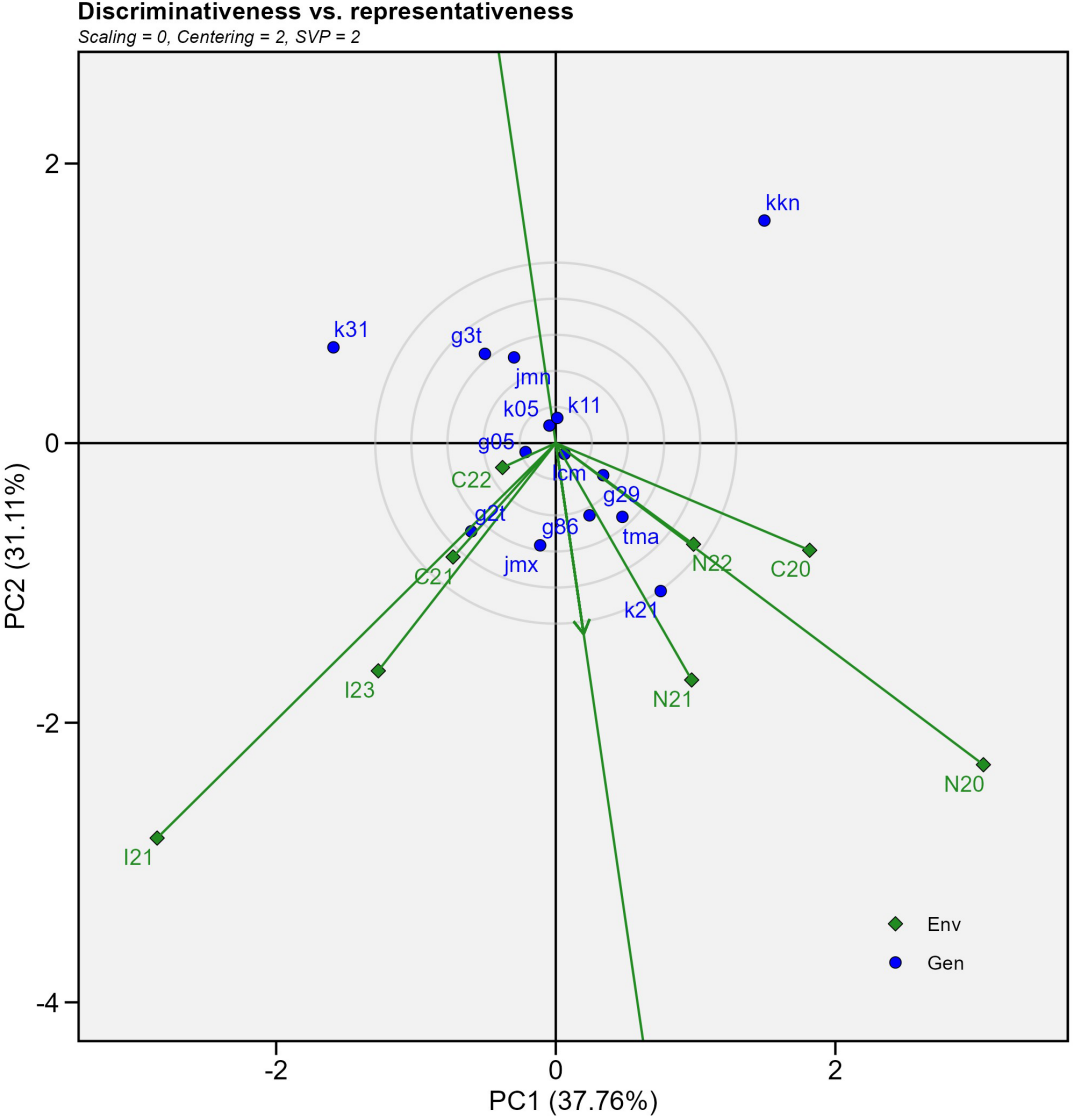
GGE biplot showing discriminating ability and representativeness of test environments. Environment-focused GGE biplot indicating the discriminating ability and representativeness of test environments. Longer environment vectors indicate greater discriminative power, while smaller angles with the average environment axis indicate higher representativeness.

The discriminativeness vs. representativeness view of the GGE biplot (**Figure 7**) was used to evaluate the relative usefulness of test environments. In this biplot, the length of an environment vector reflects its discriminating ability, whereas the angle between the vector and the AEA indicates representativeness. Environments I21 exhibited the longest vectors, indicating the highest discriminating ability among the test environments. Environment N20 also showed a relatively long vector, suggesting a strong capacity to differentiate genotypes. In contrast, environments such as C22 and C21 were positioned closer to the origin, indicating comparatively lower discriminating power. Regarding representativeness, N21 formed the smallest angle with the AEA, indicating that it was the most representative environment of the overall target conditions. Environments positioned at wider angles relative to AEA, including I21 and I23, were less representative. Therefore, I21 was highly effective for distinguishing genotype performance, whereas environments N21 best reflected the average environmental conditions across the testing network.

#### 3.4.4 Ranking of test environments

**Figure 8:**
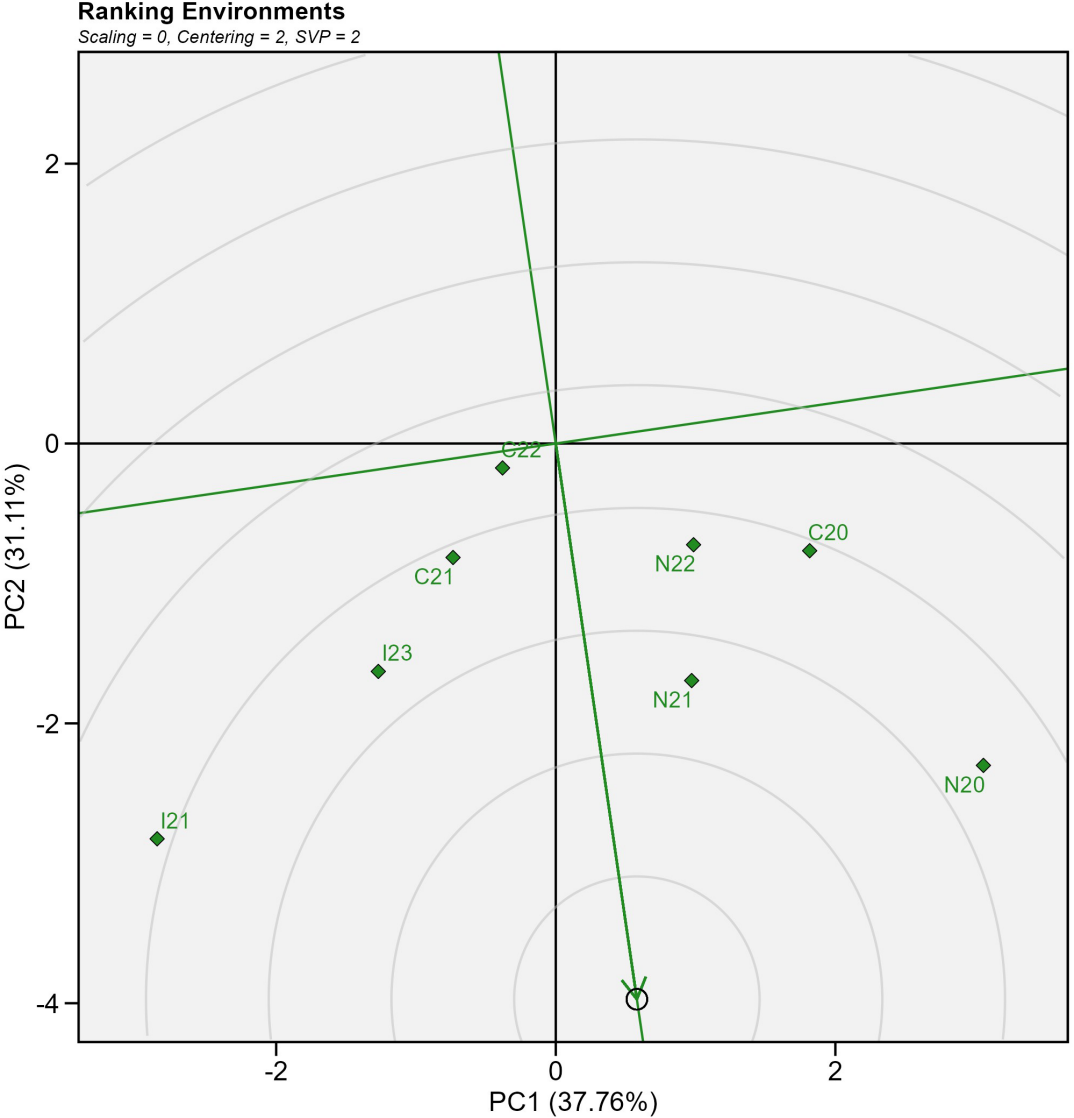
GGE biplot ranking test environments for yield. Environment-focused GGE biplot based on the first two principal components (PC1 and PC2), showing the discriminating ability and representativeness of test environments. Environments with longer vectors from the origin are more discriminating, while those forming smaller angles with the average environment axis are more representative of the overall target environment.

**Figure 8** evaluates test environments relative to an ideal environment, represented by the center of the concentric circles. An ideal environment combines high discriminating ability with strong representativeness. Environments located closer to the center of the concentric circles are considered more desirable for genotype evaluation. Among the environments, N21 was positioned closest to the ideal environment, indicating a favorable balance between discriminating ability and representativeness. Although I21 exhibited strong discriminating ability, it was positioned farther from the ideal environment due to lower representativeness.

#### 3.4.5 Ranking of genotypes for yield and stability

**Figure 9:**
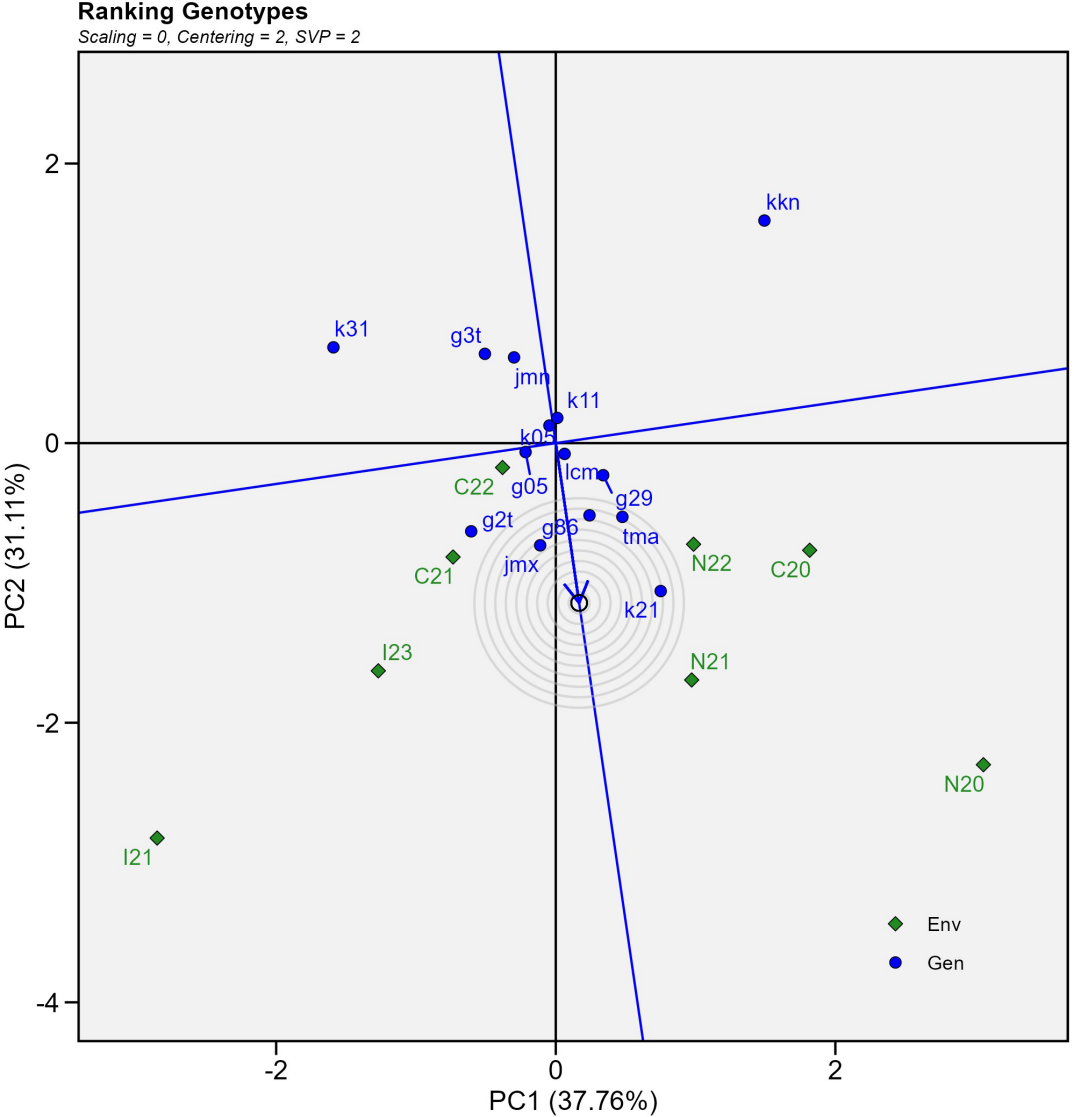
GGE biplot ranking genotypes for mean yield and stability. Genotype-focused GGE biplot based on the first two principal components (PC1 and PC2), showing genotype performance and stability across environments. Genotypes closer to the average environment axis and with shorter projections onto the stability axis exhibit greater yield stability, whereas genotypes positioned further along the positive direction of the axis show higher mean yield.

**Figure 9** evaluates genotypes relative to an ideal genotype, represented by the center of the concentric circles. The ideal genotype combines high mean biomass yield with high stability across environments. Genotypes positioned closer to the center of the concentric circles are considered more desirable, as they exhibit both superior mean performance and greater stability. In this study, jmx and k21 were located closest to the ideal genotype, indicating a favorable balance between yield and stability. Genotype kkn was in the opposite direction of the AEA arrowhead, indicating below-average yield despite showing strong interaction effects. Genotypes positioned farther from the ideal point also exhibited lower overall desirability when both mean performance and stability were considered.

#### 3.4.6 Relationships among test environments

**Figure 10:**
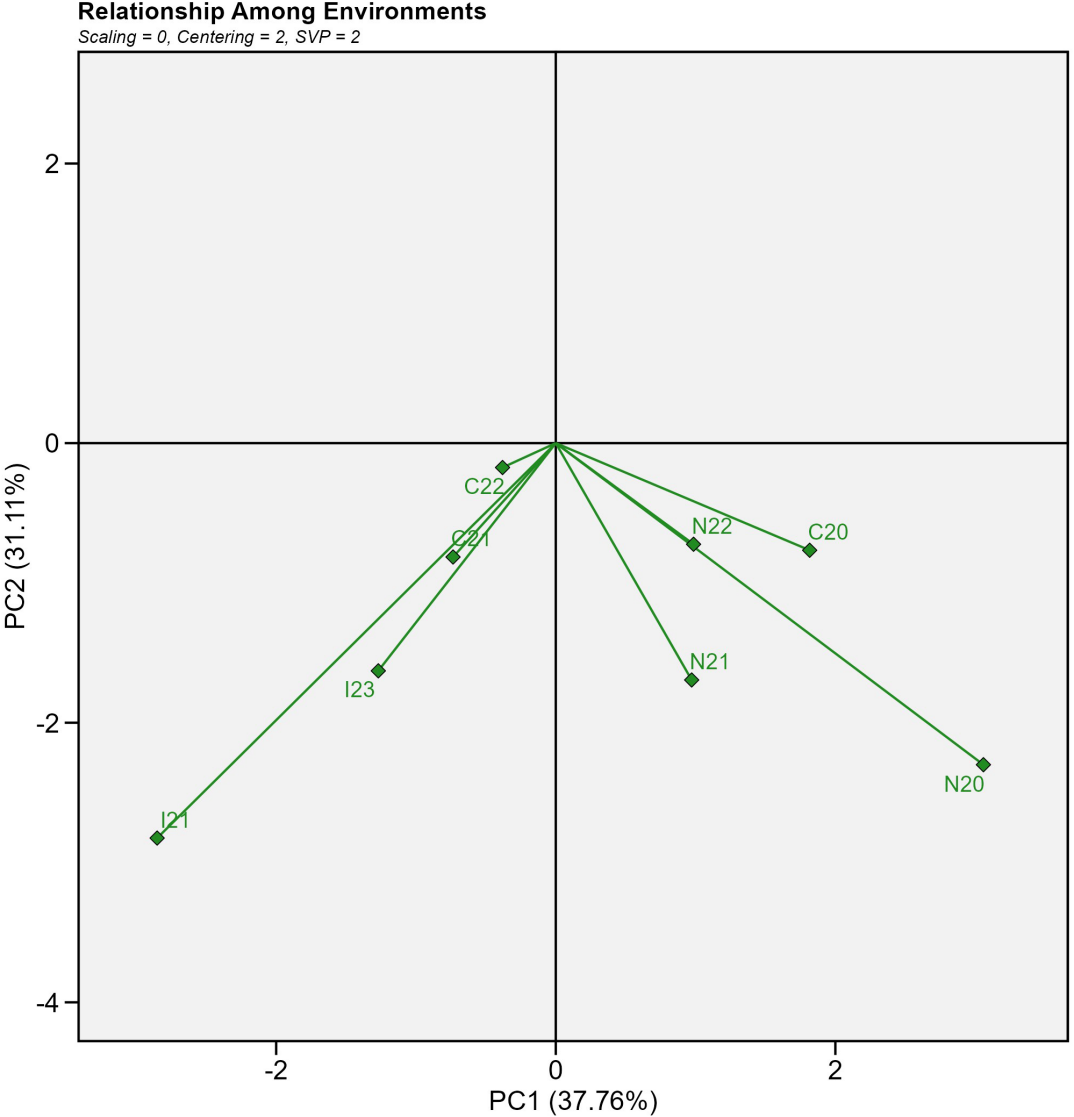
GGE biplot showing relationships among test environments for yield. Environment-focused GGE biplot based on the first two principal components (PC1 and PC2), showing the relationships among environments. The angle between environment vectors indicates the correlation between environments, where acute angles indicate positive correlations, obtuse angles indicate negative correlations, and near-right angles indicate weak or no correlation.

The environment-focused GGE biplot illustrating relationships among test environments is shown in **Figure 10**, which depicts the correlation structure among environments based on the angles between their vectors. Acute angles between environment vectors indicate positive correlations, obtuse angles indicate negative correlations, and near-right angles suggest weak or negligible correlations. Environments N22 and C20 formed relatively acute angles, indicating positive correlation and similar genotype performance patterns. Likewise, C21 and C22 were closely aligned, suggesting comparable environmental responses. In contrast, environments such as I21 formed wider angles relative to N20 and C20, indicating weaker or contrasting genotype responses. The dispersion of vectors across different quadrants indicates variability in environmental conditions across location-year combinations. This indicates that growing conditions were quite different across the testing sites, which is consistent with the significant G×E found in the AMMI analysis (**Table 5**).

### 3.5 Genotype BLUP estimates

**Figure 11:**
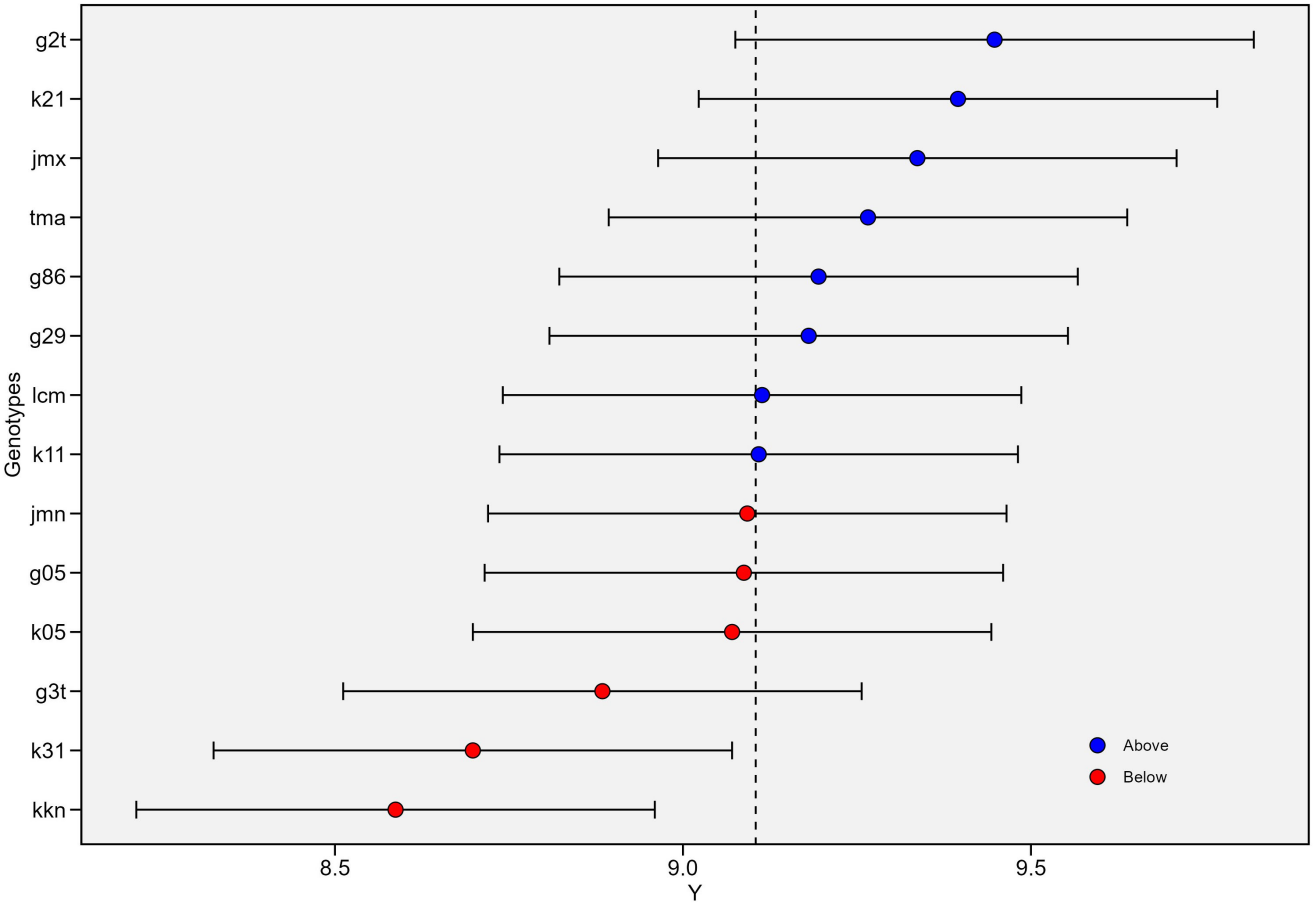
Genotype BLUPs for annual yield across environments. BLUPs of genotype annual yield (Y) estimated from a mixed-effects model treating genotypes and G×E as random effects. Points represent genotype BLUP means, horizontal bars indicate associated uncertainty intervals, and the vertical dashed line denotes the overall mean yield. Genotypes positioned to the right of the mean show above-average performance, whereas those to the left show below-average performance.

Variance component estimates and likelihood ratio tests confirmed significant genotypic and G×E effects under the mixed-effects model (**Table 2**). **Figure 11** presents BLUPs for genotype performance across environments. The mean genotype BLUPs ranged from approximately 8.6 to 9.4 Mg ha^-1^ (**Figure 11**), indicating moderate variability in predicted mean yield among genotypes after adjusting for environment and interaction effects. Genotypes g2t, k21, jmx, and tma had the highest BLUPs, showing superior overall performance. In contrast, kkn and k31 had the lowest BLUPs, reflecting lower predicted mean yield. Several genotypes, including g29, g86, g05, k11, and lcm, had BLUPs near the overall mean, suggesting intermediate performance. The distribution of BLUPs supports the meaningful genotypic differences after adjusting for environmental heterogeneity. Residuals from the mixed-effects model showed approximate symmetry around zero (**Supplementary Figure S2**), indicating no major deviation from normality assumptions. The Q–Q plot further confirmed that standardized residuals closely followed the theoretical normal distribution, with only minor deviations at the tails (**Supplementary Figure S3**).

### 3.6 Probability-based genotype ranking

**Figure 12:**
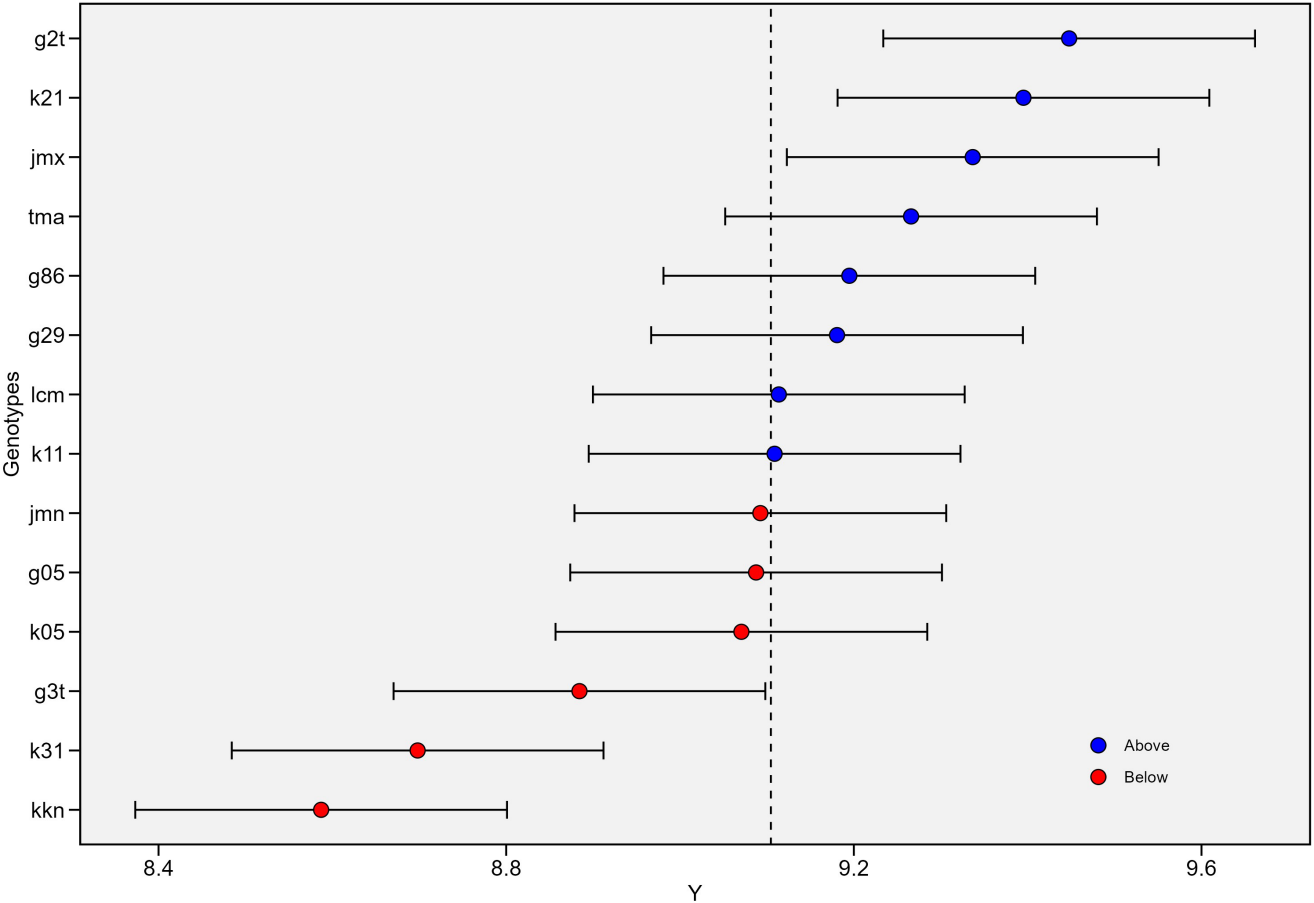
Probability-based ranking of genotype BLUPs for annual yield. Genotype BLUPs for annual yield (Y) displayed with probability-based classification relative to the overall mean. Points represent genotype BLUP means with associated uncertainty intervals, and the vertical dashed line indicates the overall mean yield. Genotypes shown in blue have a higher probability of performing above the overall mean, whereas genotypes shown in red have a higher probability of performing below the mean. Inverted ordering highlights relative rankings among genotypes.

The probability-based ranking of genotypes (**Figure 12**) further evaluated the likelihood of each genotype being among the top-performing entries across environments. Genotypes g2t and k21 showed the highest probabilities of superior performance, consistent with their elevated BLUP estimates. Genotype jmx also demonstrated a relatively high probability of ranking among the top performers. In contrast, kkn and k31 exhibited the lowest probabilities of superior performance, aligning with their lower BLUP values. Genotypes with intermediate BLUP estimates displayed moderate probability values, indicating stable but not outstanding performance. Overall, the probability-based ranking corroborated the BLUP-based mean performance and provided additional confidence in identifying broadly superior genotypes across environments.

### 3.7 Integrated yield and stability parameters

Mean yield values in **Table 6** represent estimated marginal means obtained from the ANOVA model. This genotype performance showed relatively small differences among several entries. Multiple comparison analysis using Tukey’s HSD revealed that many genotypes shared overlapping significance groups, indicating that several apparent ranking differences were not statistically significant. For example, genotypes g2t and k21 formed the highest statistical group, whereas several intermediate genotypes (e.g., k05, g05, jmn, k11, and lcm) were not significantly different from each other. Similarly, the lowest-yielding genotypes kkn and k31 belonged to the same statistical group.

**Table 6:** Mean biomass yield, standard error, 95% confidence intervals, and Tukey’s HSD grouping of 14 tall fescue genotypes evaluated across eight environments.

Genotype	Mean yield (Mg ha^-1^)	SE	95% CI	Group
g2t	10.22	0.18	9.69–10.74	a
k21	10.20	0.18	9.67–10.72	a
jmx	10.05	0.18	9.52–10.57	ab
tma	9.97	0.18	9.45–10.49	ab
g29	9.83	0.18	9.31–10.35	ab
g86	9.83	0.18	9.30–10.35	ab
lcm	9.71	0.18	9.18–10.23	abc
k11	9.69	0.18	9.16–10.21	abc
jmn	9.64	0.18	9.12–10.17	abc
g05	9.63	0.18	9.11–10.16	abc
k05	9.62	0.18	9.09–10.14	abc
g3t	9.28	0.18	8.76–9.81	bc
k31	8.92	0.18	8.39–9.44	c
kkn	8.89	0.18	8.37–9.41	c

Groups sharing the same letter are not significantly different at α = 0.05.

Because multiple genotypes were statistically indistinguishable in mean yield, numerical rankings derived from individual stability or adaptability indices occasionally differed. Integrated stability and adaptability indices (**Table 3**), therefore, provided additional criteria for differentiating genotype performance. Stability parameters, including Shukla’s stability variance, ASV, and WAASB, identified lcm, k05, and k11 as relatively stable genotypes, whereas kkn and k31 exhibited the highest interaction and instability values across indices. Yield–adaptability metrics derived from BLUPs, including RPGV, HMRPGV, and Lin and Binns’ superiority index (Pi), identified g2t, k21, and jmx as superior performers combining higher yield with acceptable stability.

To integrate yield performance and stability into a unified selection criterion, the YWSI was applied. The YWSI combined BLUP-predicted yield with AMMI-based stability to provide a composite ranking of genotype performance (**Table 7**). Based on YWSI values, g2t, jmx and k21 were among the highest-ranked genotypes for combined yield and stability. However, because several genotypes showed overlapping significance groups for mean yield, caution is warranted when interpreting differences among closely ranked genotypes. Overall, genotype rankings derived from YWSI were consistent with graphical (AMMI and GGE) and BLUP-based analyses, providing a practical criterion that accounts for both mean performance across environments and stability.

**Table 7:** Yield weighted stability Index (YWSI) ranking of 14 tall fescue genotypes evaluated across eight environments, based on BLUP-predicted biomass yield (Mg ha^-1^) and AMMI stability value (ASV).

Genotype	C20	C21	C22	I21	I23	N20	N21	N22	ASV	Rank C20	Rank C21	Rank C22	Rank I21	Rank I23	Rank N20	Rank N21	Rank N22	Rank ASV	YWSI	Rank YWSI
g2t	6.42	13.24	7.4	8.26	5.43	11.33	17.06	12.01	1.14	1	1	1	1	5	10	2	5	10	36	1st
jmx	6.08	12.68	6.81	7.85	5.86	11.99	16.79	11.89	0.47	5	3	4	2	1	4	6	8	5	38	2nd
k21	6.24	12.19	6.78	7.54	5.77	12.67	17.31	12.08	1.23	2	11	5	5	2	1	1	3	11	41	3rd
tma	6.16	12.43	6.65	7.33	5.46	12.12	16.95	12.08	0.68	4	6	9	7	3	3	3	3	7	45	4th
g29	5.92	12.33	6.66	7.19	5.3	11.82	16.85	12.17	0.47	7	7	7	9	7	5	5	1	5	53	5th
lcm	5.88	12.31	6.46	7.33	5.14	11.42	16.88	12.09	0.12	9	8	12	7	12	8	4	2	1	63	6th
g86	5.88	12.59	6.66	7.61	5.28	12.38	16.38	11.61	0.77	9	4	7	4	8	2	11	11	9	65	7th
g05	5.7	12.49	6.75	7.48	5.22	11.58	16.54	11.47	0.37	12	5	6	6	9	6	10	12	4	70	8th
k05	5.98	12.2	6.47	7.12	5.43	11.21	16.73	11.91	0.2	6	10	11	11	5	11	7	7	2	70	8th
k11	5.87	12.31	7.08	7.1	5.17	11.38	16.65	11.92	0.27	11	8	2	12	11	9	8	6	3	70	8th
jmn	6.22	13.17	6.92	6.77	5.1	10.65	16.65	11.79	1.3	3	2	3	13	13	13	8	9	12	76	11th
g3t	5.53	12.01	6.53	7.13	5.2	10.78	16.14	11.69	0.68	13	12	10	10	10	12	12	10	7	96	12th
k31	4.38	11.82	6.43	7.79	5.45	10.55	15.68	10.88	2.25	14	13	13	3	4	14	14	14	13	102	13th
kkn	5.9	11.6	6.36	5.04	3.92	11.58	16	11.38	2.47	8	14	14	14	14	6	13	13	14	110	14th

BLUP-predicted biomass yields for individual environments (C20, C21, C22, I21, I23, N20, N21, and N22) are expressed in Mg ha^-1^. ASV, AMMI Stability Value; YWSI, Yield Weighted Stability Index. The YWSI integrates the rank of BLUP-predicted yield with the rank of ASV to provide a composite ranking of overall genotype performance, where lower values indicate superior combined yield and stability.

## 4 Discussions

### 4.1 Environmental influence and magnitude of G×E

The multi-environment evaluation of 14 tall fescue genotypes across eight location-year combinations revealed that environmental effects were the dominant source of variation in annual biomass (**Table 5**). This outcome is expected given the perennial growth habit of tall fescue, where temperature, rainfall, harvest frequency, and local field management strongly influence biomass accumulation. The wide yield range across environments (**Table 5**) and the contrasting yield distributions (**Supplementary Figure S3**) confirm substantial heterogeneity across the testing environments (**Figure 1**).

Iron Horse Farm (Georgia) experienced the warmest conditions with mild winters, whereas Nappan, Nova Scotia, had the coolest climate (**Figure 1**). Cornell, New York, exhibited intermediate temperature conditions between the two locations. Rainfall patterns also varied by location and year, as shown in **Figure 1**. Iron Horse Farm showed substantial interannual rainfall variability between 2021 and 2023, while Cornell and Nappan exhibited distinct seasonal rainfall distributions relative to their long-term averages. These climatic differences likely contributed to the strong environmental effect observed in the AMMI analysis.

The climatic patterns presented in **Figure 1** align with the environmental structures identified through AMMI and GGE analyses. The Nappan environments (N20, N21, and N22) clustered separately from the Iron Horse Farm environments (I21 and I23) in the AMMI2 biplot (**Figure 3**), corresponding to the cooler and warmer growing conditions of these locations, respectively. Cornell represented an intermediate climatic environment, with C20 aligning more closely with the cooler Nappan environments and C21 aligning with the warmer Iron Horse Farm environments. This pattern suggests that temperature was an important contributor to the environmental structure detected by the AMMI and GGE analyses.

Despite the predominance of environmental variance, both genotype and G×E were highly significant (**Table 5**), indicating meaningful genetic differentiation and differential responses among genotype across environments. To improve the reliability of selection decisions, G×E is analyzed using models such as AMMI and GGE. These models help identify both broadly adapted genotypes that perform consistently across environments and specifically adapted genotypes that perform best in particular environments. The G×E component, although smaller than the environmental main effect, was large enough to cause changes in ranking (crossover interaction); therefore, the use of AMMI and GGE models is helpful.

### 4.2 AMMI analysis and biomass yield stability across environments

The AMMI model effectively summarized the interaction structure, with the first two IPCAs capturing most of the biologically relevant G×E signal. In agreement with the foundational work of Zobel et al. (1988), AMMI was particularly suitable here because multi-environment yield data often contain both strong main effects and meaningful G×E patterns that are not well characterized by ANOVA alone. In this study, AMMI not only confirmed the presence of G×E but also decomposed it into a small number of interpretable interaction axes (IPCA1 and IPCA2), allowing clear visualization of crossover responses and genotype stability.

Genotypes with small IPCA scores demonstrated reduced sensitivity to environmental fluctuations, reflecting greater yield stability and broader adaptation. Such genotypes are particularly valuable for forage systems spanning diverse climatic zones, where consistent biomass production is often prioritized over peak performance in a single environment. In contrast, genotypes with large interaction scores exhibited stronger environmental responsiveness, suggesting specific adaptation and potential utility in targeted production regions. The presence of crossover interaction highlights that genotype superiority is environment-dependent, reinforcing the limitation of relying solely on grand mean yield for selection decisions. For perennial species like tall fescue, integrating interaction patterns with mean performance enables more strategic cultivar development - either for broader adaptation or for deployment within defined/targeted environmental clusters.

The AMMI2 biplot (**Figure 3**) illustrates the environmental gradient identified by the AMMI analysis. Environments N20, N21, N22, and C20 were on the positive side of IPCA1, while I21, I23, and C21 were on the negative side. This split matches the climate differences shown in **Figure 1**, with Nappan being cooler and Iron Horse Farm warmer. The middle-range climate at Cornell likely led to C20 and C21 being placed differently within these groups. These results suggest that temperature played a key role in the environmental pattern captured by the first interaction principal component, although rainfall variability may also have influenced genotype responses across environments.

In this study, AMMI was used to quantify and interpret G×E and to evaluate genotype stability using interaction principal component axes and AMMI-derived stability parameters. Although AMMI analysis was effective in characterizing G×E, it provides limited information for delineating mega-environments, the representativeness and discriminating power of test environments, identifying the ideal test environment, and the relationships among environments. These complementary aspects were further explored using GGE biplot analysis.

### 4.3 GGE biplot and mega-environments

GGE biplot complemented the AMMI approach by focusing on the combined effect of genotype and G×E rather than interaction effects alone. In addition to evaluating genotype performance, GGE biplots helped delineate mega-environments, identify winning genotypes within environmental clusters, and evaluate test environments for their representativeness and discriminating ability. The GGE biplot explained a substantial proportion of genotype plus G×E variation (**Figure 4**). The “which-won-where” biplot (**Figure 5**) delineated specifically two mega-environments with particular winner genotypes - k21 is the best performer in the mega-environment formed by Nova Scotia environments and C20, while g2t is the superior genotype in the mega-environment formed by Iron Horse Farm environments and C21.

The mega-environment delineation also closely corresponded to climatic differences among locations (**Figure 1**). The mega-environment comprising N20, N21, N22, and C20 represented relatively cooler growing conditions, whereas the mega-environment comprising I21, I23, and C21 represented comparatively warmer conditions. The superior performance of k21 and g2t within these respective mega-environments suggests adaptation to cooler and warmer climatic conditions, respectively. Rainfall variability and seasonal precipitation patterns may have further contributed to differences in genotype responses across these environments. Therefore, rather than targeting a single broadly adapted genotype, breeding programs may adopt region-specific cultivar deployment strategies.

A similar study in sugarcane showed that genotype performance and stability varied across locations and years, and that GGE biplots were useful for identifying location groupings, discriminating test environments, and identifying stable cultivars for selection (Todd et al., 2018). The identification of distinct mega-environments in the present study is consistent with findings reported by Missaoui et al. (2024), who also observed clear mega-environment delineation and environment-specific winner populations using GGE biplot analysis. In their study, different tall fescue populations were superior in Athens versus Blairsville environments, reflecting strong crossover interaction patterns.

In the present study, k21 and g2t emerged as winners across different mega-environments, underscoring the importance of region-specific genotype recommendations rather than selection based solely on overall mean biomass yield. These interpretations should be considered in the context of variation attributable only to the first two principal components, as additional variation not captured by the GGE biplot may also contribute to adaptation characteristics. The mean vs. stability analysis (**Figure 6**) showed that genotypes such as jmx and k21 combined above-average yield with acceptable stability. These same genotypes also approached the ideal genotype position in **Figure 9**, suggesting broader adaptation across environments. Similarly, genotypes such as kkn combined lower yield with greater instability and would therefore be less desirable for cultivar recommendation.

### 4.4 Evaluation of test environments

An additional advantage of GGE biplot analysis is that it facilitates assessment of test environments for their representativeness and discriminating ability, identification of the ideal environment, and relationships among environments. **Figures 7**-**10** present the environment-focused GGE analysis emphasizing the importance of testing environments. Environments such as I21 exhibited high discriminating ability but lower representativeness, whereas N21 combined strong representativeness with moderate discriminating power (**Figure 7**). An ideal test environment should balance these two attributes. N21, being closest to the ideal environment (**Figure 8**), may serve as a primary testing site for preliminary genotype evaluation. Highly discriminating environments like I21 are valuable for identifying performance differences among genotypes, but may not fully represent the average target production environment. The correlation structure among environments (**Figure 10**) showed that certain location-year combinations shared similar genotype response patterns, while others differed substantially. The positive associations observed between environments N22 and C20 align with their relatively similar temperature profiles shown in **Figure 1**. These findings suggest that environments with similar temperature and rainfall patterns tended to produce comparable genotype responses, thereby contributing to the correlation structure observed in the GGE biplot. The knowledge of correlated and non-correlated environments may help optimize future trial designs and improve the efficiency of genotype evaluation across locations and years.

### 4.5 Mixed model BLUP analysis and selection

The mixed-effects model with genotype and G×E as random effects provided shrinkage-based estimates as a robust alternative to fixed effect means under partially unbalanced data (**Table 1**). The LRT in **Table 2** shows that the interaction effect contributes much more strongly to model fit than genotype alone, while confirming significant effects of genotype and G×E under the REML framework. Compared to raw means, BLUP estimates narrowed the yield range. Genotypes g2t, k21, jmx, tma, g86, and g29 consistently ranked higher under BLUP (**Figure 11**). Probability-based ranking (**Figure 12**) provided an additional layer of inference by quantifying the likelihood of superior performance while strengthening decision-making by incorporating uncertainty rather than relying solely on point estimates. For tall fescue breeding programs, such integrated metrics enhance confidence in selecting genotypes for advancement.

### 4.6 Integrated yield and stability

Although significant genotype effects were detected, mean yield differences among several entries were relatively small, and multiple comparison analysis indicated overlapping significance groups. Although YWSI effectively integrates multi-location yield performance and stability into a single criterion, it is best used for comparative decision support rather than as a definitive indicator of significant superiority among closely ranked entries. Similar patterns have been reported in multi-environment trials of perennial forage crops, where environmental variability often reduces the magnitude of detectable differences among genotypes. Such statistical overlap emphasizes the complexity of selecting superior genotypes based solely on mean performance.

Because the mean yield alone was insufficient to clearly discriminate among several entries, stability and adaptability metrics provided additional insight into genotype performance. Stability parameters such as Shukla’s variance and AMMI-based indices have been widely used to quantify genotype contribution to G×E and consistency across environments. In contrast, BLUP-based adaptability measures such as HMRPGV and superiority index (Pi) have been shown to improve identification of broadly adapted genotypes in multi-environment trials. These considerations have led to the development of selection approaches that combine yield and stability into a single selection criterion to better exploit G×E in crop performance trials (Kang, 1993).

The integration of yield and stability into a single composite metric through the YWSI provided a balanced framework for selection. Composite indices that jointly consider productivity and stability have been proposed to facilitate breeder-oriented decision making in variable environments. A recent study applying multi-environment stability analysis in switchgrass integrated AMMI-derived stability measures with environment-specific BLUP predictions through a YWSI, enabling simultaneous evaluation of productivity and stability (Irfan et al., 2026). That study demonstrated the effectiveness of combining mixed-model prediction and interaction-based stability assessment for identifying broadly and specifically adapted genotypes in perennial biomass crops. The present study extends this integrative framework to tall fescue, further supporting the utility of composite yield–stability indices in perennial forage breeding. The integration of BLUP-predicted yield and AMMI-derived stability within the YWSI provides a practical approach for identifying genotype groups that demonstrate superior performance and acceptable stability. The positive concordance of YWSI with AMMI, GGE, and BLUP-based results further establishes its utility as a comparative decision-support tool for multi-environment yield evaluation. The consistency of YWSI rankings with AMMI, GGE, and BLUP-based results further supports its applicability in multi-environment evaluation. Such integrative approaches provide breeders with practical tools to balance productivity and stability in multi-environment trials, thereby improving the reliability of genotype recommendations across variable environments (Kang, 1993).

### 4.7 Implications for tall fescue breeding

The study underscored the environmental heterogeneity as the primary determinant of tall fescue biomass yield. The significant G×E suggests that genotype selection must incorporate stability analysis to avoid overestimating performance in favorable environments. Genotypes k21, jmx, and g2t consistently exhibited high mean biomass yield and stable performance across multiple analyses. These genotypes represent strong candidates for broad adaptation. On the other hand, genotypes with specific adaptation may be deployed in the defined mega-environments. Overall, integration of AMMI, GGE, and BLUP approaches enhances the identification of resilient, high-yielding genotypes suitable for diverse agroecological regions. This multi-prong analysis framework helps in more precise cultivar recommendations, breeding efficiency, and sustainable tall fescue forage production.

### 4.8 Limitations

In this study, tall fescue genotypes were evaluated based on annual biomass yields across three locations: Iron Horse Farm (Georgia), Cornell (New York), and Nova Scotia (Canada). While yield variation among these locations can largely be attributed to differences in weather patterns and soil conditions, an important factor that also influences yield is the number of harvest cuts per year. This variability in harvest frequency complicates direct comparisons of environmental productivity. Therefore, caution must be exercised when identifying the most discriminating environment. While our results allow us to discuss the most ideal genotypes for broad adaptation or stability, it is less straightforward to determine a single “most ideal” environment for selection.

The stability parameters and genotype rankings presented in this study are based on evaluations conducted across eight environments; therefore, interpretations should be confined to these specific conditions. While the results offer valuable insights into genotype adaptation and stability, rankings among closely performing genotypes may change if future evaluations incorporate additional environments. In addition, endophyte association is a factor influencing tall fescue yield performance, stability, and environmental adaptation (Bacon et al., 1977; Bouton et al., 1993; Repussard et al., 2014). The present study, however, has not included this aspect. Future studies integrating endophyte characterization with multi-environment stability analyses may further improve the understanding of tall fescue genotype adaptation and yield performance across different environments.

## 5 Conclusions

The multi-environment evaluation of 14 tall fescue genotypes across eight location-year combinations demonstrated that environmental effects were the predominant source of variation in annual biomass yield, while genotype and G×E effects were also highly significant. The presence of crossover interaction indicates that genotype ranking varied across environments, underscoring the importance of evaluating both stability and adaptability in cultivar selection. The combined use of AMMI and GGE biplot analyses effectively characterized interaction patterns, supported mega-environments delineation, and facilitated identification of broadly adapted and specifically adapted genotypes. Genotypes k21, jmx, and g2t consistently exhibited favorable yield and stability across multiple analytical approaches and were among the most promising genotypes identified in this study. Other genotypes exhibited stronger interaction effects, suggesting potential utility within defined environmental clusters rather than across the entire testing network.

Mixed-model analysis using REML-based BLUPs provided shrinkage-based performance estimates under partially unbalanced data and reinforced genotype rankings obtained from AMMI and GGE approaches. The YWSI, developed to integrate BLUP-predicted yield and AMMI-based stability, offered a composite selection framework that accounted simultaneously for productivity and stability across environments. The consistency of YWSI rankings with graphical and mixed-model analyses further supports its utility as a practical decision-making tool in multi-environment trials. Overall, integrating AMMI, GGE, BLUP, and YWSI methodologies provides a comprehensive framework for interpreting G×E and improving selection efficiency in tall fescue breeding programs. This multi-model strategy enhances identification of high-yielding, broadly adapted genotypes while also enabling targeted deployment within specific mega-environments to optimize forage productivity across diverse agroecological conditions.

## Data Availability

The raw data supporting the conclusions of this article will be made available by the authors, without undue reservation.

## Supplementary material

Supplementary Figure S1 Histograms of annual yield across eight test environments, each defined by a specific location and year. Yield range and variability differ among environments, highlighting environmental variation in multi-environment trials. These plots supported exploratory data assessment before mixed-model analysis.

Supplementary Figure S2 Overall residual distribution from the mixed-effects model. The histogram displays residuals from the mixed-effects model used to estimate genotype BLUPs for annual yield. The distribution is approximately symmetric around zero, suggesting no substantial deviation from model assumptions.

Supplementary Figure S3 Quantile-quantile (Q-Q) plot of standardized residuals from the mixed-effects model used to estimate genotype BLUPs for annual yield. The residuals closely align with the theoretical normal distribution, exhibiting only minor deviations at the tails. This pattern indicates that the normality assumptions of the model are not substantially violated.

Supplementary Table S1 AMMI genotype and environment means with corresponding IPCA scores.
