# Supplementary material for: Forage yield stability of tall fescue genotypes across eight location-year combinations in the United States and Canada
Source: Front Plant Sci. 2026 Jul 23;17:1870120. doi: 10.3389/fpls.2026.1870120 (PMC13442827; doi:10.3389/fpls.2026.1870120)
Supplement: Supplementary file 1 [file SupplementaryFile1.pdf]

Supplementary Material

1 Supplementary Figures and Tables

1.1 Supplementary Figures

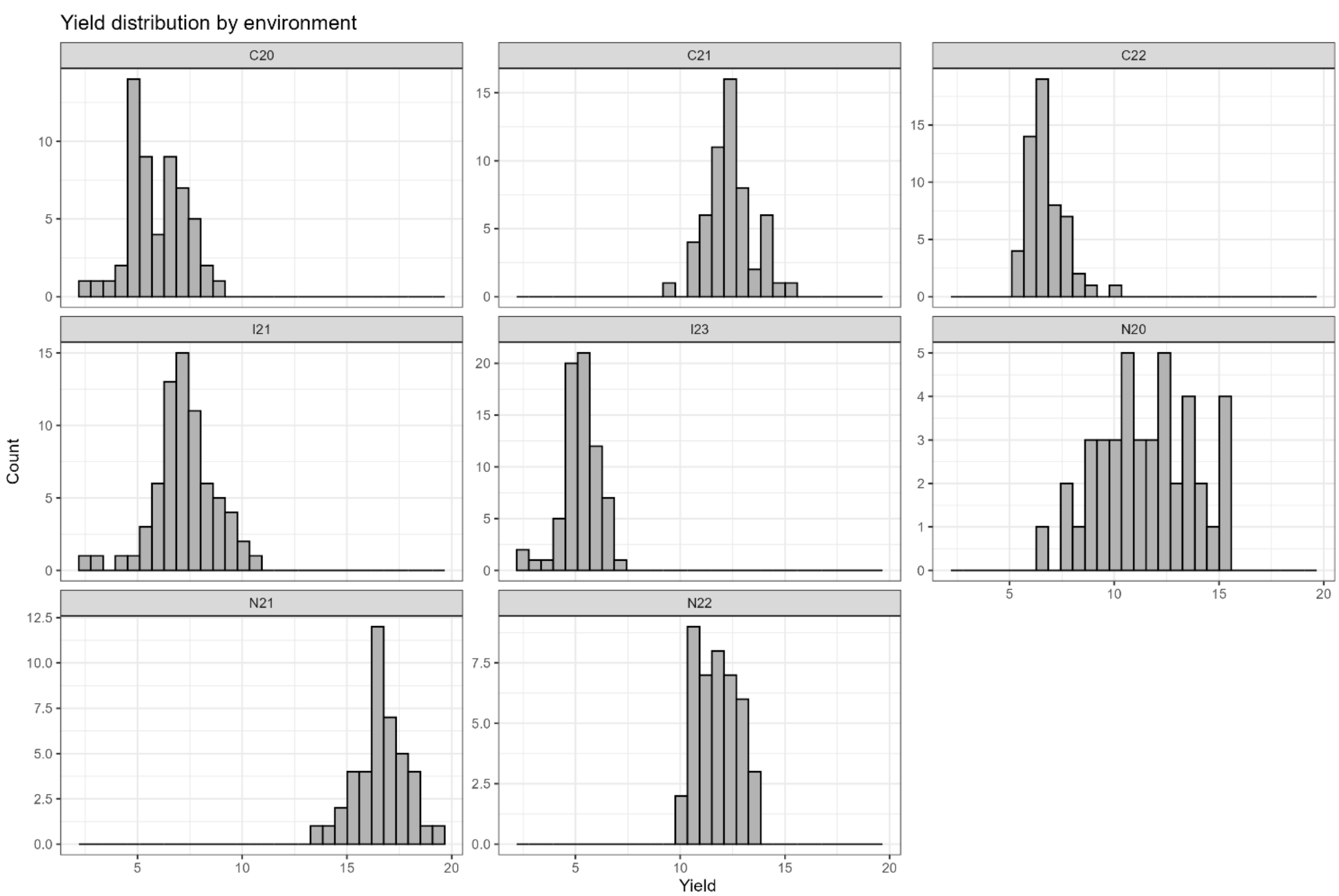

Supplementary Figure S1. Histograms of annual yield across eight test environments, each defined by a specific location and year. Yield range and variability differ among environments, highlighting

environmental variation in multi-environment trials. These plots supported exploratory data assessment before mixed-model analysis.

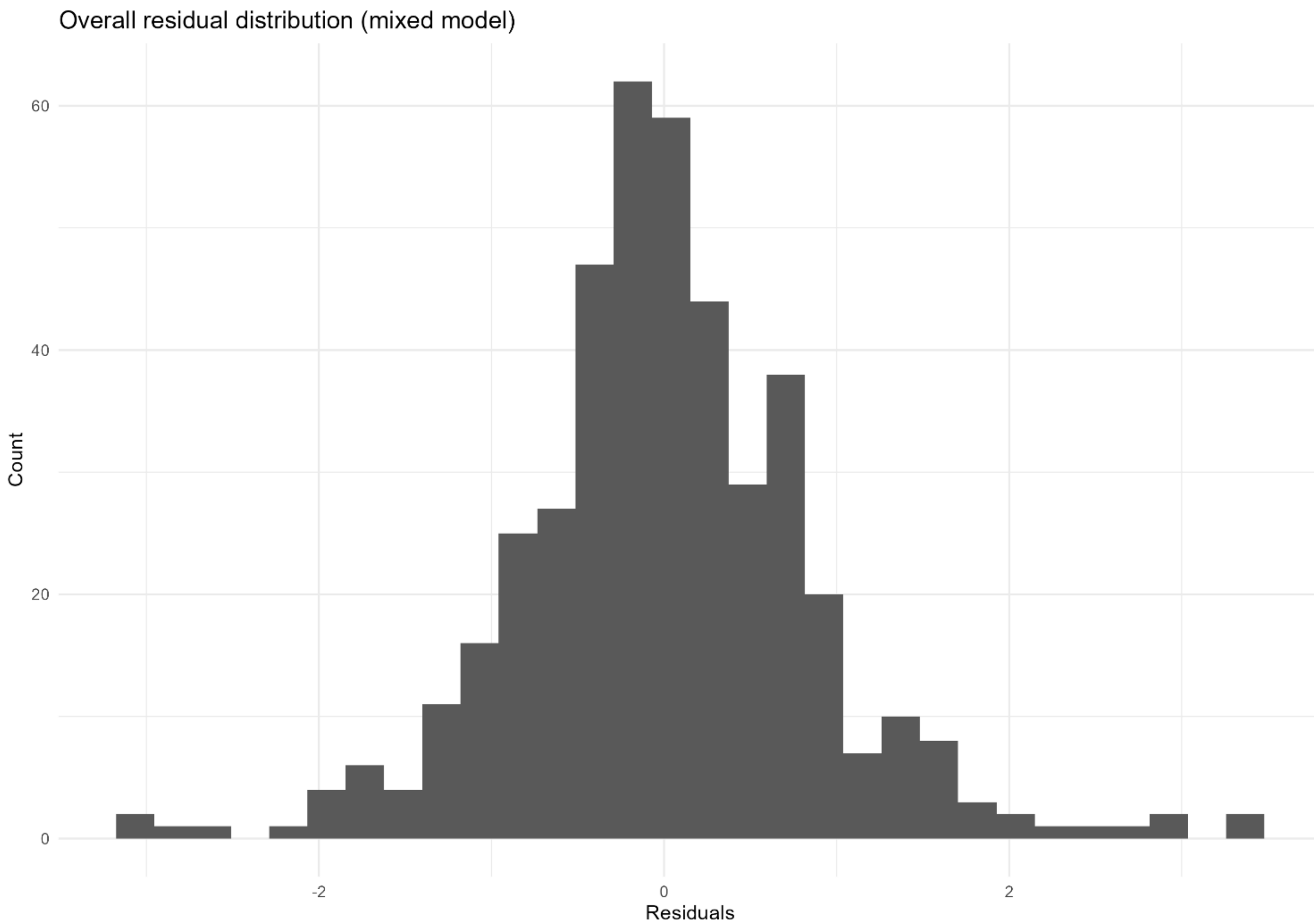

Supplementary Figure S2. Overall residual distribution from the mixed-effects model. The histogram displays residuals from the mixed-effects model used to estimate genotype BLUPs for

annual yield. The distribution is approximately symmetric around zero, suggesting no substantial deviation from model assumptions.

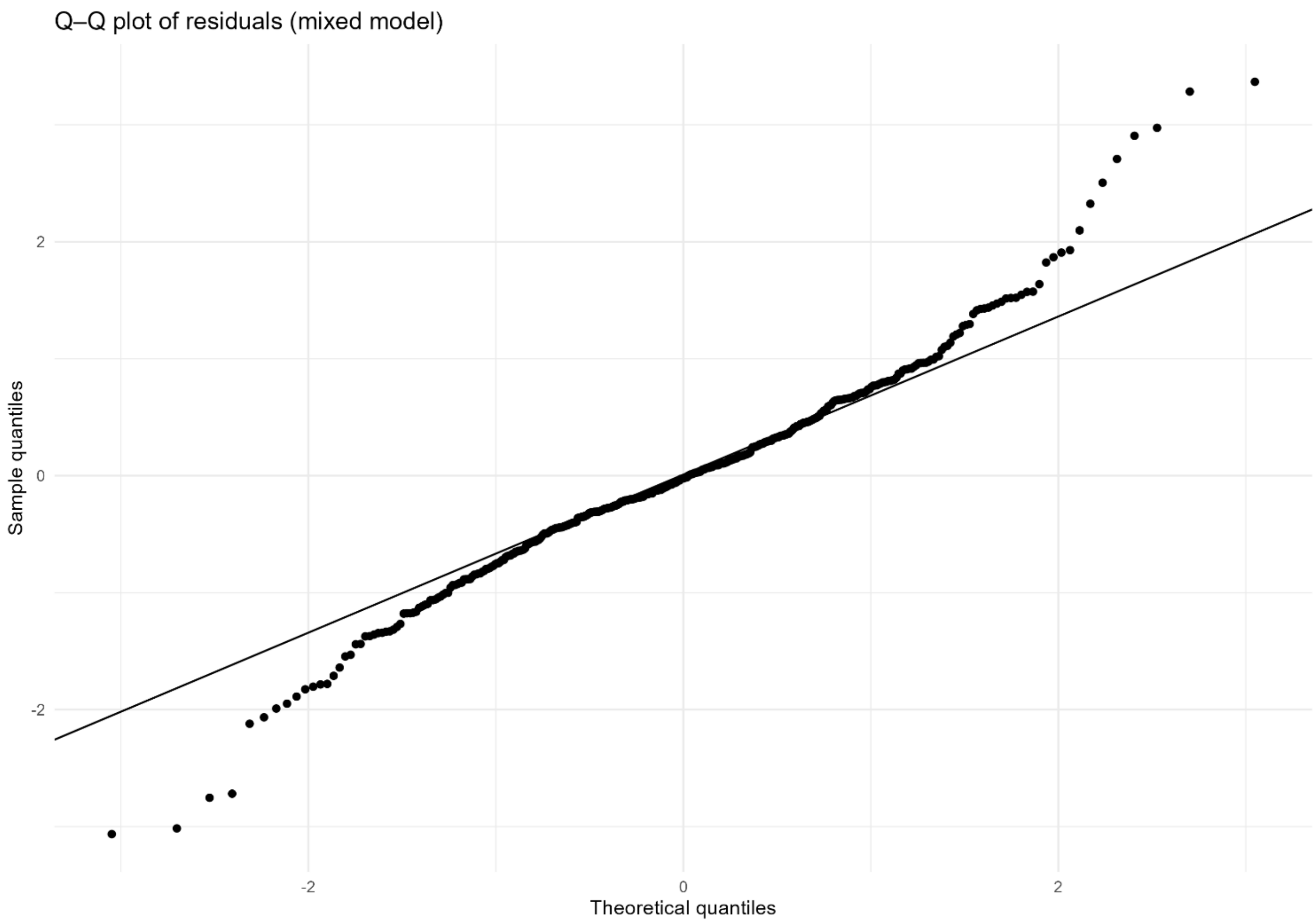

Supplementary Figure S3. Quantile-quantile (Q-Q) plot of standardized residuals from the mixed-effects model used to estimate genotype BLUPs for annual yield. The residuals closely align with the theoretical normal distribution, exhibiting only minor deviations at the tails. This pattern indicates that the normality assumptions of the model are not substantially violated.

1.2 Supplementary Table

Genotype or Environment	S.N.	Mean	IPCA1	IPCA2
Genotype				
g2t	1	9.71	-0.6	-0.53
k21	2	9.56	0.56	0.79
jmx	3	9.52	-0.16	0.39
tma	4	9.35	0.37	0.29
g86	5	9.26	0.17	0.71
g29	6	9.2	0.27	0.1
lcm	7	9.1	0.05	-0.09
k11	8	9.1	0.02	-0.26
g05	9	9.09	-0.19	0.18
jmn	10	9.09	-0.22	-1.25
k05	11	9.05	-0.03	-0.19
g3t	12	8.76	-0.38	-0.24
k31	13	8.52	-1.3	0.52
kkn	14	8.14	1.45	-0.41
Environment				
N21	1	16.62	0.32	-0.18
C21	2	12.38	-0.4	-0.74
N22	3	11.78	0.38	-0.38
N20	4	11.53	1.24	1.31
I21	5	7.25	-1.42	0.72
C22	6	6.71	-0.2	-0.48
C20	7	5.87	0.74	-0.69
I23	8	5.27	-0.66	0.44

Supplementary Table S1. AMMI genotype and environment means with corresponding IPCA scores.
